# Activated Corrosion and Recovery in Lead Mixed-Halide Perovskites Revealed by Dynamic Near-Ambient Pressure X-ray Photoelectron Spectroscopy

**DOI:** 10.1021/jacs.5c00668

**Published:** 2025-02-27

**Authors:** Michel De Keersmaecker, Paul Dietrich, Mounib Bahri, Nigel D. Browning, Neal R. Armstrong, Erin L. Ratcliff

**Affiliations:** † School of Materials Science and Engineering, Laboratory for Interface Science of Printable Electronic Materials, Georgia Institute of Technology, 771 Ferst Drive NW, Atlanta, Georgia 30332, United States; ‡ Department of Chemistry and Biochemistry, The University of Arizona, 1306 E. University Way, Tucson, Arizona 85721, United States; § SPECS Surface Nano Analysis GmbH, Voltastraße 5, Berlin 13355, Germany; ∥ Department of Mechanical, Materials and Aerospace Engineering, University of Liverpool, 506 Brodie Tower, Liverpool L69 3GQ, U.K.; ⊥ School of Chemistry and Biochemistry, Georgia Institute of Technology, 901 Atlantic Drive NW, Atlanta, Georgia 30332, United States

## Abstract

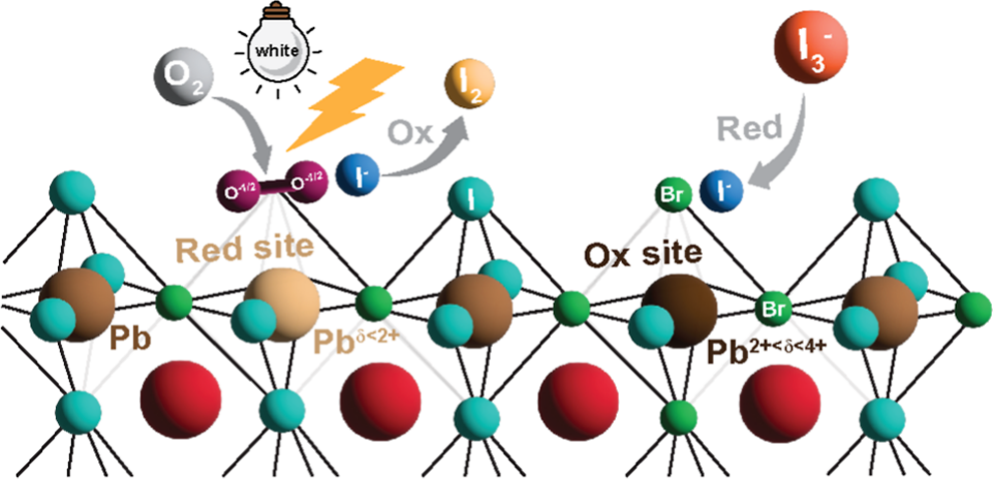

Herein, we quantify rates of O_2_-photoactivated corrosion and recovery processes within triple cation CsFAMAPb(IBr)_3_ perovskite active layers using dynamic near-ambient pressure X-ray photoemission spectroscopy (NAP-XPS). Activated corrosion is described as iodide oxidation and lead reduction, which occurs only in the presence of both O_2_ and light through photoinduced electron transfer. We observe electron density reorganization from the Pb–I bonds consistent with ligand exchange, evident from the nonstoichiometric redox change (i.e., <1 e^–^). Approximately half of the Pb centers are reduced to weakly coordinated Pb–higher oxidation number than metallic Pb–with a rate coefficient of ∼3 (±0.3) × 10^–4^ atomic percent/s. Hole capture by I^–^ yields I_3_^–^ and is accompanied by increased concentrations of near-surface bromides, hypothesized to be due to anion vacancies and/or oxidation of mobile iodide resulting from ion demixing. Activated corrosion is found to be quasi-reversible; initial perovskite stoichiometry slowly recovers when the O_2_/light catalyst is removed, postulated to be due to mobile halide species present within the film below XPS sampling depth. Small deviations in near-surface composition (<2%) of the perovskite are used to connect reaction rates to quantified, near-band edge donor and acceptor defect concentrations, demonstrating two energetically distinct sites are responsible for the redox process. Collectively, environmental flux and rate quantification are deemed critical for the future elucidation of chemical degradation processes in perovskites, where rate-dependent reaction pathways are expected to be very system dependent (environment and material).

## 1 Introduction

Understanding the chemical factors that dictate long-term stability in metal halide perovskite thin films is critical in the optimization of fully commercialized printable energy conversion, display and optoelectronic platforms,^1−3^ X-ray detectors,^4−6^ and photodetectors.^7−9^ The scientific community generally agrees instabilities are associated with a variety of defects within the perovskite crystal lattice.^10−15^ Degradation-coupled defects include interfacial interactions,^16−18^ phase instabilities,^19^ and nanoscale heterogeneities.^20^ A number of environmental stressors have been reported, such as temperature fluctuations,^21^ exposure to ambient gases,^22,23^ and fluctuations in energy-dependent photon flux (from X-rays^24−26^ to UV–visible regions^27^). Yet, a conclusive mechanism remains elusive due to complexity and multilength scale effects. For example, exposure to above bandgap light cleaves weak Pb–I bonds within PbI_6_^4–^ octahedra to form “anion defects” and undercoordinated or weakly coordinated (Pb_WC_) sites, “cation defects,” or even more extensive displacements in the form of substitutional, interstitial and/or antisite defect states.^12^ Migrating and segregated halide anions along grain boundaries have been reported to reduce phase and structural stability.^28−30^ At conditions far from equilibrium (i.e., under large electric fields), degradation is exacerbated by subsequent halide oxidation which prohibits recovery.^31^ Most reactive defects are hypothesized to originate during solution deposition due to incomplete nucleation of intermediate colloids (such as PbI_2_-solvent adducts^32^ or other impurities^33^) and formation of off-stoichiometry domains (PbI_2_ inclusions,^34−36^ grain boundaries^37^ and interfaces^38^) during crystallization. This large distribution of grain sizes, orientations and crystalline phases creates energetic gradients and preferred reactive regions for external stressors.^39^

Ultimately, reaction pathway identification necessitates understanding which parameters affect reaction rates and are critical to further optimization of perovskite photoactive layers used within many optoelectronic technology platforms. In this work, we report activated corrosion reaction rates in the near-surface region of mixed triple cation halide perovskite active layers (PALs) using dynamic near-ambient pressure X-ray photoelectron spectroscopy (NAP-XPS) in the presence of 2 mbar dry O_2_ gas. Dynamic (NAP-)XPS historically refers to the time-dependent quantification of chemical species within the near-surface region, a slight distinction from in situ or operando XPS.^40−42^ As a comparative study with the broader perovskite literature, we consider a perovskite composition (FA_0.79_MA_0.16_Cs_0.05_)Pb(I_0.87_Br_0.13_)_3_ (or Cs_.05_FA_.79_MA_.16_) that is prototypical of top performing PALs in solar cells.^43−45^ The Cs_.05_FA_.79_MA_.16_ formulation has also been the focus of our (spectro)electrochemical approach to understand band edge and defect energetics and defect quantification.^13−15^ For the stoichiometric composition (Cs_.05_FA_.79_MA_.16_), we have demonstrated electrochemically reactive cation defects within ca. 100 meV of the conduction band minimum (CBM) at concentrations near 10^15^ cm^–3^, and reactive anion defects within ca. 100 meV of the valence band maximum (VBM) at concentrations near 10^14^ cm^–3^; these reported defect levels are typical for perovskites in high-performing solar cells.^15^

Using carefully chosen oxygen partial pressure and X-ray fluence, we are able to determine rate coefficients of activated corrosion in the near-surface regions of Cs_.05_FA_.79_MA_.16_ films exposed to O_2_ and light (white light + X-ray). In the absence of O_2_, no reaction is observed over the course of the experiment. A subsequent control shows minimal chemical reaction in the absence of X-ray/light with O_2_ exposure. Collectively, these two controls rationally suggest O_2_ must catalyze photoinduced electron transfer at the perovskite surface, although we cannot directly track the photoinduced electron transfer reaction between a perovskite and O_2_ yielding superoxides, O_2_^•–^ or even peroxides, O_2_^2–^.^46−49^ Shifts in core-level binding energies (BE) for Pb 4f, I 3d, and Br 3d photoelectrons demonstrate to be quite sensitive to small changes in the Pb coordination environment (nominally corner-sharing PbX_6_^4–^ octahedra, where X = I^–^, Br^–^) and are used to support our conclusions. We also utilize a subset of UHV-based XPS measurements to determine Auger parameters for relevant species as a second self-check. Specifically, we can detect anion displacement (and oxidation), supporting prior hypotheses that the superoxide can serve as a weak ligand in the anion vacancy location or can undergo acid/base reactions with A-site cations to cause disproportionation, one-electron transfer, deprotonation, and nucleophilic substitution reactions.^46,50,51^ We can also resolve I^–^ in the PbX_6_^4–^ octahedra versus the oxidation product I_3_^–^ and the variable concentration of Br^–^ species as degradation and healing processes proceed. For the stoichiometric perovskite up to half of the Pb centers in the illuminated near-surface region are slowly converted from PbX_6_^4–^ octahedra to weakly coordinated Pb sites (termed Pb_WC_), which represent coordination environments with a valence state less than +2 on the Pb atom and not metallic Pb^0^ or PbI_2_. In parallel, I 3d and Br 3d spectra demonstrate mobile iodide anions undergo hole capture to form I_2_ (likely lost to vacuum) and XPS-detectable I_3_^–^, accompanied by an increase in the relative near-surface bromide concentration, consistent with “ion demixing”.^52−54^ Once UHV conditions are restored, the driving force for active corrosion, localized to the X-ray illuminated region, is removed. Pb_WC_ sites now convert to strongly coordinated Pb sites (termed Pb_SC_) defined by bromide-enriched [PbX_6_]^4–^ octahedra. Under UHV, the Pb/I atomic ratio progresses back to the stoichiometric value, with the loss of Pb_SC_ sites, consistent with diffusion limited iodide transport from regions outside the illuminated area, a process associated with “self-healing”.^55−57^

For nonstoichiometric PALs derived from 2% FAI-rich perovskite precursor solutions,^13−15^ the anodic and cathodic reaction rates of the active corrosion process are increased, consistent with excess I^–^ acting as a hole trap to form I_3_^–^ and with the presence of deep donor traps that energetically align with the O_2_/O_2_^•–^ reaction.^58^ In contrast, for 2% FAI-deficient/PbI_2_-rich films, the degradation mechanism is considered different as also suggested in literature,^34−36^ consistent with our reported lowered anion defect densities,^15^ which slow down the loss of photoinduced iodide. These NAP-XPS studies suggest that compositional and interfacial modifications proposed to enhance perovskite stabilities should be amenable to characterization by this dynamic approach and that NAP-XPS studies can be generalized to environmental stressors affecting performance in a variety of optoelectronic platforms. Likewise, rate determination as a function of material combination and environmental stressors provides a viable pathway toward mechanistic understanding and advancing the development of state-of-the-art metal halide perovskite materials.

## 2 Results and Discussion

### 2.1 Experimental Design Considerations and Control Experiments

**Figure 1:**
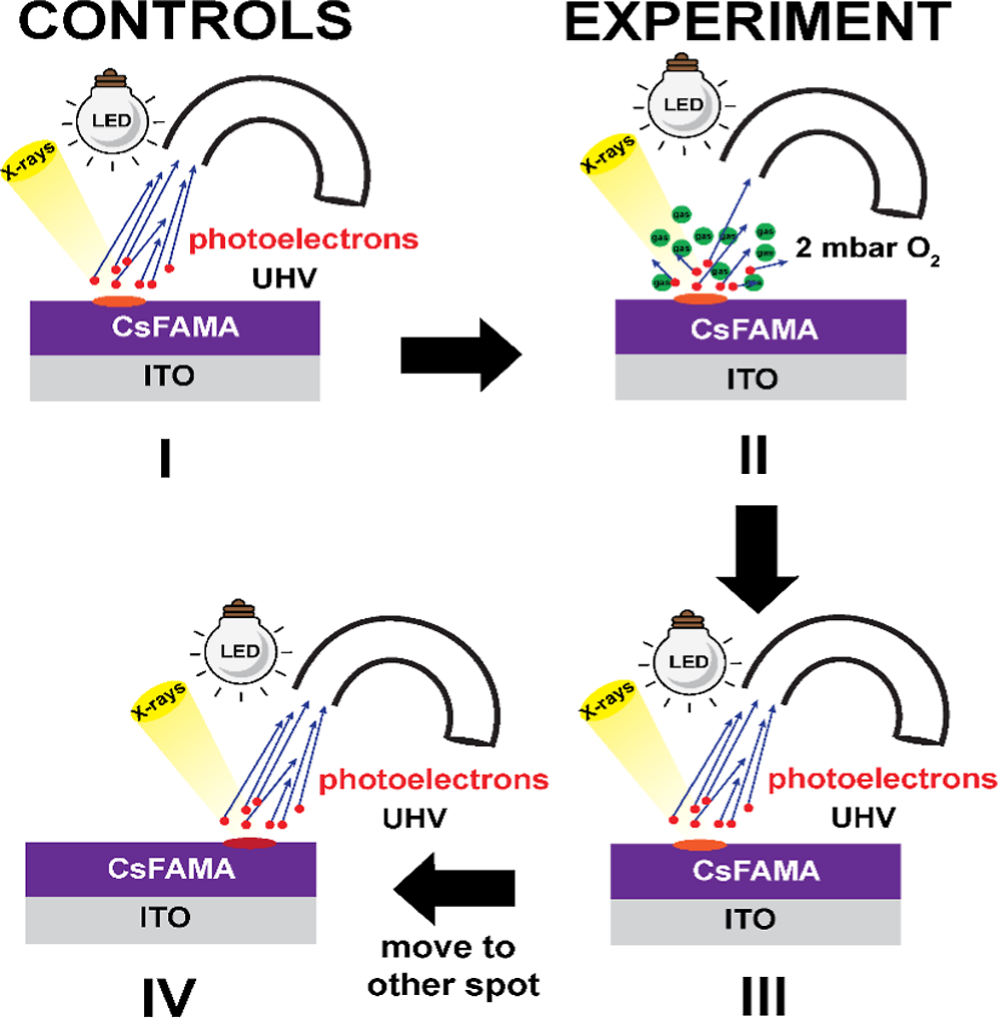
Schematic of the NAP-XPS experiments and controls for determination of rates of activated corrosion and recovery as a function of compositional changes in triple cation perovskites. (I) Control experiment under UHV of perovskite film before O_2_ exposure to verify surface region is stable during X-ray fluence. (II) Collection of activated corrosion rate data during O_2_ exposure (2 mbar) in combination with X-ray and white light from LED. (III) Recovery rate data collection when perovskite film is returned to UHV conditions under white light (same sample spot used for activated corrosion in II). (IV) Control experiment using a new analysis region, after O_2_ exposure alone (reference region), showing no reactivity in the absence of light.

The experimental philosophy behind these NAP-XPS experiments on device-relevant triple cation perovskite thin films is summarized in Figure 1; films have been previously characterized using X-ray diffraction (Figure S1), photoluminescence/time-resolved photoluminescence (PL, TRPL) and electrochemical defect quantification.^15^ The experimental design includes a four-step protocol used for three different perovskite formulations; the four steps consist of two experiments to capture rates of reactions bookended by two controls. A similar approach was adopted by Kot et al.^26^ for water vapor exposure using in situ NAP-XPS. Herein, the first control evaluates no changes to the near-surface region of the perovskite film when exposed to the monochromatized Al K_α_ X-ray source (1486.7 eV, illumination spot size of 0.25 × 0.25 mm^2^) under UHV conditions, indicating retention of the established perovskite stoichiometry (Experiment I). This control is critical for mechanism analysis, as it verifies the perovskite surface is stable during X-ray fluence and with white light under UHV. Recent studies of X-ray detection using perovskites, for X-rays between 1 and 2 keV suggest that electron/hole pair formation and photocurrent generation occurs with excitation at these energies, with attenuation coefficients of 10^3^ to 10^4^ cm^–1^ which suggests that ca. 50% of the X-ray fluence is absorbed within the ca. 500 nm perovskite film thickness, concentrated of course in the near-surface region.^59,60^ For white light, MAPbI_3_ demonstrates an attenuation coefficient of 10^5^ cm^–1^.^61^

Rate analysis was performed using a carefully chosen oxygen partial pressure that ensures reaction rates that allow for speciation and quantification of products using XPS, where data acquisition requires minutes per spectrum. A comparison of our experimental protocols with recently published results using NAP-XPS on different perovskite films and stressors is summarized in Table S1. Specifically, our approach uniquely captures rate coefficients for activated corrosion processes under reactive gas partial pressures that should ultimately allow for “tuning” of the stress experienced by the PAL. We note that the sampling nozzle is placed close to the excited spot to compensate for the kinetic energy dependent differential loss of inelastically scattered photoelectrons due to collisions with O_2_ gas molecules. We assume a low steady-state coverage of weakly adsorbed oxygen (O_2,ads_), that is constant versus the depth below the surface, owing to fast O_2_ diffusion within these perovskite films.^62^ Of particular note are measurements by Kot et al.^26^ for MAPbI_3_, by Lu et al.^63^ for α-FACsPbI_3_ and by Xu et al.^64^ for mixed halide MAPbI_3–*x*_Cl_*x*_ and MAPbBr_3–*x*_Cl_*x*_ systems; these studies consider bulk large area illumination with visible or UV light, where compositional changes were characterized using conventional XPS (i.e., under UHV), indicating reactions are observed without direct XPS probing in the O_2_ environment.^65^

Reaction rates are reported for perovskites undergoing activated corrosion during exposure to X-ray beam, white light and 2 mbar O_2_ (Experiment II) and during recovery (i.e., same spot with removal of O_2_; Experiment III). The second control is a sample region that was exposed to O_2_, but well outside of the illumination zone and then returned to UHV prior to X-ray analysis (Experiment IV). This control demonstrates that the perovskite does not react extensively with O_2_; i.e., O_2_ gas alone does not chemically alter the PAL nor does X-ray excitation alone induce degradation (Experiment I).

In Supporting Information Note 1 with Figures S2–S4 and Tables S2–S4, Pb 4f, I 3d, Cs 3d, Br 3d, O 1s, N 1s and C 1s high-resolution spectra at both locations for all triple cation perovskite films during Experiments I and II are analyzed to verify their relative atomic ratios. Relevant BEs for all identified species are summarized in Table 1 for brevity. The relative atomic ratios measured during step IV verify that the nonilluminated perovskite, even in the prolonged presence of O_2_, remains unaffected throughout the experiment. Additional PAL characterization has been conducted, as we have shown previously,^66,67^ using a conventional XPS system, using monochromatized Al K_α_ excitation and under UHV conditions, to confirm the peak assignments, BE shifts (I 3d_5/2_ – Pb 4f_7/2_) and “modified Auger parameters” [BE (Pb_NOO_) – BE (Pb 4f_7/2_)] to help differentiate between Pb coordination environments for solution processed PbI_2_ and PbBr_2_ thin films;^68,69^ as second internal reference for chemical assignments, which is rarely reported in metal halide perovskite literature but is well-established from transition metal studies. More experimental details are shown in the Supporting Information. In what follows, we focus on the subsequent individual experimental steps and describe their unique chemistries to propose a mechanistic vision for the active corrosion and recovery process in stoichiometric and nonstoichiometric mixed halide PALs.

**Table 1:** Overview of the Range of Pb 4f, I 3d and O 1s Core Level BE Shifts Defined in This Work^a^

core electron	chemical representation	acronym	BE range/eV
Pb 4f	corner-sharing PbX_6_^4–^ octahedra	Pb-X_6_	138.4–138.1
Pb 4f	weakly coordinated Pb atom	Pb_WC_	137.8–137.6
Pb 4f	strongly coordinated Pb atom	Pb_SC_	139.0–138.8
Pb 4f	metallic lead	Pb^0^	137.0–136.8
Pb 4f	PbI_2_ control^b^	PbI_2_	138.2
Pb 4f	PbBr_2_ control^b^	PbBr_2_	138.9
I 3d	iodides (also in octahedra)	I^–^	619.2–619.0
I 3d	triiodides and iodine	I_3_^–^/I_2_	619.9–619.7
O 1s	weakly adsorbed molecular O_2_	O_2,ads_	532.3–531.7
O 1s	weakly adsorbed O_2_^–^	O_2–,ads_	532.6–532.4
O 1s	oxygen bound to carbon	C–O	533.7–533.3

a Weakly and strongly coordinated Pb are referenced to the corner sharing Pb in the perovskite octahedron.

b Verified in Supplementary Note 6 using modified Auger parameters.

### 2.2 X-ray Induced Formation of a Weakly Coordinated Pb and the Role of Oxygen

**Figure 2:**
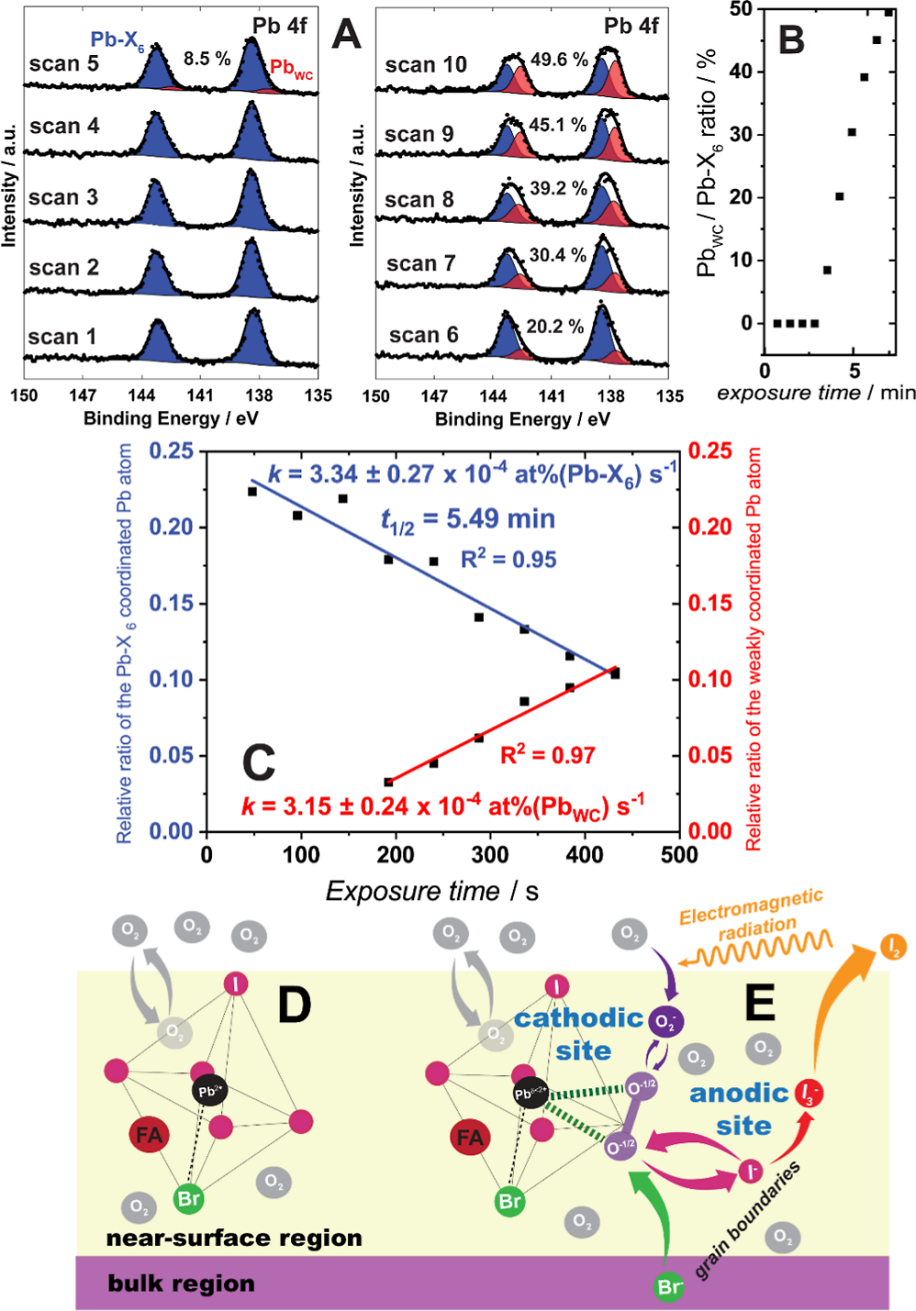
(A) Individual (scan 1–10) Pb 4f high-resolution core level spectra of a Cs_.05_FA_.79_MA_.16_ film under dry O_2_ gas exposure in combination with white light and the X-ray beam (Experiment II) in the analysis chamber with the 10-scan average Pb 4f high-resolution core level spectrum is shown in Figure S4A. The percentages indicate the ratio of weakly coordinated Pb atoms (Pb_WC_) to all Pb atoms in the XPS sampling region (ca. 10 nm). (B) The Pb_WC_-to-Pb-X_6_ ratio as a function of the exposure time to O_2_/X-ray excitation. (C) Relative atomic percentages of Pb-X_6_ (to the total atomic percentage that includes all lead, iodide, and bromide; blue, left axis) as a function of X-ray exposure time with the calculated rate coefficient (k; units atomic percentage per second). Complementary fits for the rate coefficient associated with atomic percentage of Pb_WC_ per second are also provided (red, right axis). Overview of proposed initial electrochemically coupled steps in the activated corrosion of mixed halide perovskite films in the presence of O_2_/X-ray excitation, where (D) represents the schematic of a single unit cell of hexa-coordinated Pb showing a single Br^–^ and 5 I^–^ in its coordination environment together with a steady-state concentration of weakly adsorbed O_2_; (E) depicts photoexcited electron transfer to this adsorbed O_2_, leading initially to the more strongly bound superoxide, O_2_^–.^, along with the displacement and oxidation (hole capture) of I^–^ in which ultimately I_2,g_ is formed as the complementary component of the “corrosion cell.” I_2_ is then lost to the vacuum, while I_3_^–^ remains in the grain boundaries which ultimately leads to “ion demixing” in the illuminated region.

Figure 2 provides experimental data and schematic interpretation, focusing on the Pb 4f core region under conditions of 2 mbar O_2_ in combination with white light and the X-ray beam (Experiment II in Figure 1) and we refer the reader to Table 1 for relative binding energy assignments. Figure 2A demonstrates first the Pb 4f_7/2_ core level of the PbX_6_^4–^ (or Pb-X_6_)-like octahedral environments of the perovskite at 138.2 eV. We detect the emergence of a new form of Pb with a lower binding energy (137.7 eV) at scan 5 (i.e., after ∼200 s). In XPS, binding energy shifts represent changes in polarizability of the coordination environment surrounding the photoexcited atom.^69^ Here, a shift to a lower binding energy is consistent with a weakly coordinated Pb atoms (Pb_WC_). Longer O_2_/light exposure times in Figure 2B show a considerable increase in the Pb_WC_-to-Pb-X_6_ ratio, where half of the Pb sites within the XPS sampling depth are converted to Pb_WC_ during the sampling period. We note data has been internally corrected for charge shifting by tracking BE differences for Pb_WC_ (ΔBE_I/Pb_ = I 3d_5/2_ – Pb 4f_7/2_ = 481.4 eV versus 480.9 eV for Pb in Pb-X_6_ environments; see Figure S4C). Such shifts in BE and ΔBE_I/Pb_ have been previously documented for various Pb oxides and salts, including perovskites, as the coordination strength to the Pb atom is altered.^70−72^

For elements Pb and I (see Table S3), the relative sensitivity factors (RSF) are high enough to ensure that Pb_WC_ can be detected at atomic concentrations exceeding ca. 5% of total near-surface Pb. The dynamic nature of the appearance of Pb_WC_ and the disappearance of Pb-X_6_-like coordination environments in the stoichiometric PAL is shown in Figure 2C, accompanied by complementary rate coefficients. We note that the emergence of Pb_WC_ tracks at approximately the same rate as the loss of Pb-X_6_-like species, with the latter being easier to resolve. We emphasize that these new spectral features of Pb_WC_ are not Pb^0^ (metallic lead) which is known to demonstrate an even lower Pb 4f_7/2_ BE of 136.8 eV^73^ when PbI_2_ decomposes.^74^ The relative reaction rates, both the appearance of Pb_WC_ and the disappearance of Pb in Pb-X_6_ coordination environments, expressed in atomic fraction units, are calculated to be ca. 3 × 10^–4^ atomic percent/s, which corresponds to a half-life *t*_1/2_ of 5.5 min (Table 2).

**Table 2:** Defect Densities and Rate Coefficients for the Increase of Pb_WC_ Formation and Decrease of Pb-X_6_ Loss in the PAL Films

perovskite	acceptor defect density^a^/cm^–3^	donor defect density^a^/cm^–3^	reaction pathway	k^0^/atomic fraction s^–1^	*t*_1/2_/min
Stoichiometric	1 × 10^15^	2 × 10^16^	Pb-X_6_ loss	3.34 × 10^–4^	5.5
Stoichiometric			Pb_WC_ formation	3.15 × 10^–4^	2.6
2% FAI-rich	1 × 10^18^	9 × 10^17^	Pb-X_6_ loss	2.86 × 10^–4^	4.9
2% FAI-rich			Pb_WC_ formation	2.67 × 10^–4^	3.4
2% PbI_2_-rich/FAI-deficient	none measured	2 × 10^16^	no Pb_**WC**_ formation/only Pb_**SC**_ formation		

a Electrochemically analyzed by De Keersmaecker et al.^15^

Supplementary Note 2 and Figure S5A show complementary NAP-XPS data for the I 3d region during X-ray excitation over the same period associated with experimental conditions II. We observe a new higher BE shoulder at 619.8 eV, at later times than the appearance of Pb_WC_ (after ca. 5.6 min or the time to take 7 scans), which has been previously associated with I_3_^–^ (Table 1). This observation is consistent with an expected outcome from multiple hole-capture (oxidation) events by mobile I^–^ anions (see below).^75,76^ We do not observe evidence of PbI_2_ decomposition.^77^ Br 3d spectra for these perovskite samples are shown in Figure S5B and do not show the formation of new Br species; however, the Br/I relative atomic ratios do increase with increasing X-ray fluence. We conclude that the creation of mobile iodide and oxidation to I_3_^–^ is accompanied by “ion demixing” in the near-surface XPS sampling region. We note that bromide oxidation is not observed under oxidative stress as observed in literature.^78−80^

Given the low RSF for O 1s XPS lines in Table S2 and extended chemistry between carbon and oxygen species, characterization of adsorbed forms of oxygen is much more challenging, as described in Supporting Information Note 3 and Figures S6–S9. The as-received samples show a weak and broad O 1s envelope (532–533 eV) at the beginning of the analysis, which we fit with two peaks. The first we assign to weakly adsorbed O_2_ (lower BE) and the second to oxygen bound to carbon (C–O; higher BE) (Table 1). By scan 4, the O 1s peaks have shifted to even lower BE, which is verified based on the narrow O 1s core level peaks of molecular O_2_(g) due to its paramagnetic nature defined by 1 eV splitting,^81^ while at the same time demonstrating a drastic increase in overall oxygen content as seen in Figure S7. We propose this BE shift is a result of the formation of superoxides (O_2_^–.^), most likely adsorbed at sites formerly filled by iodides (Table 1). The O 1s high-resolution core level spectra of both control experiments (see Figure 1) in Supporting Information Note 3 and Figures S8 and S9 demonstrate no BE shifts and very slow changing oxygen content.

Superoxide formation at the illuminated surface causes side reactions with the C–O species to form C–O based anions and radicals, consistent with a lower BE O 1s photoemission peak.^46^ This is clearly a dynamic process that evolves across the individual scans depending on the amount of adsorbed O_2_^–.^ present at that time in conjunction with the O_2_ gas in the chamber. This hypothesis is informed by XPS studies of superoxide or peroxide states formed in electron-rich metal–organic frameworks,^82^ at potassium-doped graphite surfaces,^83^ and at metal surfaces, but final determination of the state of adsorbed O_2_, and the extent to which peroxide states are also formed, is limited by its low sensitivity and complicated chemistry.^84,85^

To summarize our findings, we suggest a dynamic active corrosion process of coupled, unequilibrated reactions described in Figure 2D,E for the nearly exponential formation of Pb_WC_ states under saturated O_2_ conditions (i.e., reduction) coupled with formation of I_3_^–^ states as the result of 2-electron oxidation of I^–^ through hole capture from the excited perovskite.

#### 2.2.1 Initiation Step: Molecular Oxygen Adsorption

In the first step in Figure 2D, weak adsorption of molecular O_2_^47,48,62,86,87^ is assumed to reach a constant steady-state coverage at the perovskite film surface and exposed grain boundaries. The diffusion coefficient of O_2_ in PALs with organic A-site cations has been determined to be in the range 10^–9^ to 10^–7^ cm^2^ s^–1^,^62,86^ which is unlikely to create concentration gradients across the PAL. The weak coordinate-covalent bonding between Pb–I and Cs–Pb has been found to become unstable during above bandgap excitation,^88^ whereby iodide anions tend to migrate to the grain boundaries and leave behind anion vacancies within the grains.

#### 2.2.2 Reduction Step: Electron Transfer of Photogenerated electron(s) to Generate O_2_^–.^and Displace I^–^

In the presence of molecular O_2_, these anion vacancies can be filled with adsorbed O_2_ to form an excited oxygen-intercalated perovskite.^47−49^ Photogenerated electron transfer from the perovskite to O_2,ads_ is then predicted to initiate superoxide formation, O_2_^–.^, and possibly peroxides, O_2_^2–^, as summarized in the proposed sequential (reversible) scheme in Figure 2D.^50^ These reactions are predicted to displace iodide from Pb-X_6_^4–^-like environments at the surface and within grain boundary regions, producing neutral O_2_^–.^/Pb_WC_ species in Figure 2E. In Supporting Information Note 4 and Figure S10–S13, we discuss the high-resolution N 1s and C 1s core level spectra to explain why the decomposition of organic cations under the presence of X-ray irradiation/superoxide, previously defined as the most typical degradation pathway,^62,89^ is highly unlikely. Briefly, we postulate that the organic cations (proton donors) are not observed to participate in the active corrosion (i.e., redox) reaction due to the absence of a nucleophile in the analysis chamber; (i.e., water/proton acceptor to attract protons from the organic).

#### 2.2.3 Oxidation Step: Hole Transfer to Mobile I^–^

**Figure 3:**
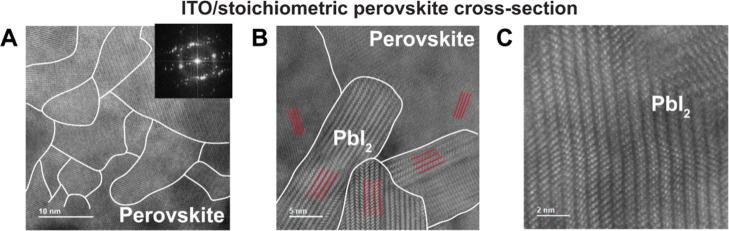
High resolution, low electron dose STEM-HAADF images of a typical stoichiometric perovskite thin (<100 nm) film that demonstrate (A) an array of grains with average diameters of ca. 10–20 nm, (B) the misalignment (red lines) in the grains with the formation of PbI_2_ inclusions, even though this PAL shows good performance in solar cells and (C) perfect aligned, single crystal-like grains. The white lines drawn on the STEM-HAADF image represent the grain boundaries in the perovskite film. FAI-rich versions of this same PAL also show lower concentrations of these PbI_2_ inclusions. Inset in A is the fast Fourier transform of the image.

Photogenerated holes are available to be neutralized via proximate sequential electron transfer events from I_gb,mobile_^–^. Oxidized I^–^ might be lost to the vacuum in the form of I_2_, but the XPS in Figure S5A suggests a bound I_3_^–^ in Figure 2E.^58,79,90^ The presence of these mobile iodides in stoichiometric perovskite films is explained in Figure 3, which demonstrates high resolution, low electron dose STEM-HAADF images of mixed halide perovskite lamella. Figure 3A shows that, even for top performing PALs, arrays of misaligned grains in the stoichiometric perovskite regions are observed with average diameters of ca. 10–20 nm, as has been seen in other STEM/HRTEM investigations,^91−93^ suggesting that unreacted ions in high local concentrations could settle between the grains at their boundaries (indicated with white lines). The corresponding fast Fourier transform in Figure 3A confirms that these active layers consist of arrays of misaligned but coherent crystalline grains (or polycrystalline structure) with a high concentration of grain boundaries.^93^ The misalignment within the grains is easily seen in the STEM-HAADF images by focusing on crystalline PbI_2_ inclusions in Figure 3B (red lines).^94^ These inclusions are formed within the highly performing stoichiometric perovskite film because of incomplete insertion of A-site cations and their accompanying iodide counterion during perovskite crystallization.^95^ Further magnification in Figure 3C demonstrates that these PbI_2_ inclusions are sharper because they have more ordered structures with higher atomic number elements (as HAADF is a *Z*-contrast imaging technique) compared to the perovskite crystal which includes random octahedra of iodide and bromide, as well as various organic–inorganic cations including lighter atomic number elements. Their role in mitigating corrosion processes is discussed further below.

Critical to the proposed a mechanism is that activated corrosion reactions can occur concurrently as spatially separated events (a “corrosion cell”) at cathodic sites of weakly coordinated lead/superoxide and iodide-enriched anodic sites as shown in Figure 2E, a concept that has been widely described in studies of active corrosion mechanisms.^96−98^ Under these assumptions the reactions detailed in these XPS data are predicted to be first order in total X-ray photon dose as deduced in Supporting Information Note 5 and Table S5. As discussed further below, upon return to UHV conditions we anticipate that these adsorbed superoxide species will be removed, providing for coordination sites to be filled by either Br^–^ or I^–^ migrating from below surface regions of the perovskite.

### 2.3 Oxygen Removal Leads to the Loss of Weakly Coordinated Pb and the Introduction of a Stronger Coordinated Form of Pb

**Figure 4:**
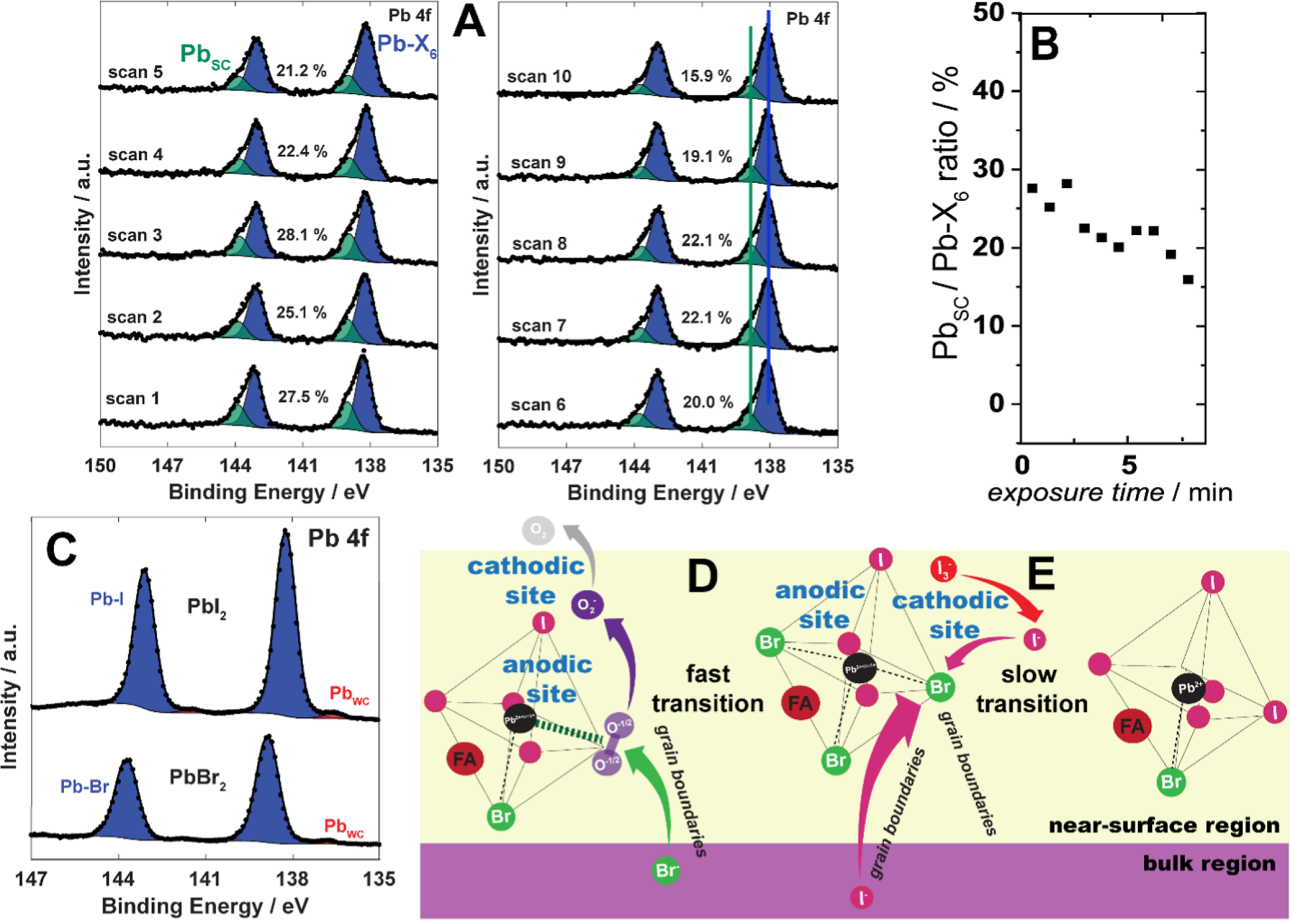
(A) Individual (scan 1–10) Pb 4f high-resolution core level spectra of a stoichiometric Cs_.05_FA_.79_MA_.16_ film after return to UHV conditions under white light (step III) in the measuring chamber. The percentages indicate the ratio of Pb_SC_ atoms to all Pb atoms. (B) The Pb_SC_/Pb-X_6_ ratio as a function of the exposure time to UHV/X-ray excitation. (C) High-resolution Pb 4f core level spectra and for a PbI_2_ and PbBr_2_ film exposed to an inert atmosphere to demonstrate the BE shift in the coordination environment of the Pb atom. (D) Overview of critical steps in the activated degradation of mixed halide perovskite films associated with return to UHV/X-ray excitation conditions, including (D) the displacement (oxidation to O_2_) of superoxide ligands, and because of the local depletion of I^–^, enrichment of Br^–^ as a coordinating ligand in the near surface region referred to as “ion demixing”; (E) the gradual return to the stoichiometric condition as a result of I^–^ diffusion into the X-ray illuminated near-surface region referred to as “self-healing”.

Figure 4A shows the individual Pb 4f core level spectra obtained after the degradation reaction has occurred and the chamber has returned to UHV conditions without moving the sample position. Interestingly, Pb_WC_ decreases below the XPS detection limits, accompanied by the formation of more strongly coordinated Pb species (or Pb_SC_) (BE of Pb 4f_7/2_ = 138.8 eV—green peaks in Figure 4A and Table 1), relative to the corner-sharing [PbI_6_]^4–^ octahedra (BE of Pb 4f_7/2_ = 138.2 eV—blue peaks in Figure 4A). Figure 4B demonstrates a slow continuous decrease in the Pb_SC_/Pb-X_6_ ratio, suggesting that the Pb_SC_ species are converted back to the original Pb-X_6_ coordination under influence of X-ray and white light excitation only. This specific BE shift for these newly formed Pb_SC_ species is consistent with the formation of Pb–Br_*x*_I_6–*x*_^4–^ (bromide-enriched) environments with *x* > 1 as indicated in Figure 4C, which compares shifts in the Pb 4f core level spectra for pure PbI_2_ and PbBr_2_ films. Further evidence is given in Supporting Information Note 6 and Figure S14 using both Pb 4f core level spectra and Pb N_7_O_4,5_O_4,5_ Auger lines for pure PbI_2_ and PbBr_2_ films. This BE shift compared to the PbI_2_ film is explained by the dipole moment difference along the Pb–I bond in PbI_2_ (μ_e_ = 4.3 D) and the Pb–Br bond (μ_e_ = 5.0 D) in PbBr_2_,^99^ as well as the polarizability of the bulk material.^69^ Additionally, Table S6 confirms the I/Pb ratio decrease and Br/Pb increase, which is expected when bromide enrichment takes place. All Pb_WC_/Pb-X_6_ and Pb_SC_/Pb-X_6_ ratios of the stoichiometric perovskite during all steps described in Figure 1 are shown in Figure S15.

#### 2.3.1 Rationale for Bromide-Enriched Near-Surface Region

The reason for the bromide enrichment process can be rationalized by a combination of redox and ligand chemistry. First, from a redox perspective, photogenerated holes will preferentially oxidize iodide over bromide anions, due to the lower standard reduction potential of iodide;^53^ excess bromide is expected to remain within the grain boundaries of the illuminated region. Second, the superoxide radical (i.e., a conjugated base of weak acid) is a better leaving group in ligand exchange reactions than the bromide nucleophile (i.e., a conjugated base of a strong acid). Under UHV conditions, the steady-state O_2gas_/adsorbed O_2_/O_2_^–.^/Pb_WC_ condition is eliminated, which further promotes bromide enrichment. Hence, at the start of the experiment, the illuminated spot has been locally depleted of iodides and is now infused with bromide-enriched octahedra.

#### 2.3.2 Partial Pb–I_6_ Recovery

Under UHV conditions and white light in Figure S16, the I 3d region shows a slow loss of I_3_^–^ over the duration of ca. 10 min of analysis time. This suggests triiodide ions are slowly reduced back to I^–^ via electron capture, which can then slowly reform Pb-X_6_-like octahedra explaining the self-healing properties within perovskites as demonstrated in Figure 4E. A second possible pathway for self-healing is to consider the surrounding perovskite material that can act as a “reservoir” of replacement iodide to feed the repair of the damaged spot. This latter process is supported by data in Figure S17A, which shows a clear drop in the excess of Br_gb,mobile_^–^, examining the Pb/Br ratio. Figure S17B demonstrates a slight increase in the Pb/I ratio while the decrease in the Pb/O ratio in Figure S17C explains the loss of adsorbed O_2_ under UHV. However, all Pb-X_6_^4–^-like coordination environments are not completely restored as seen in Figure 4E, suggesting that the bromide enrichment in the near-surface region and the concurrent ion demixing process is not fully reversible in the time course of these experiments due to the slow mass transport of the iodide anions from the “reservoir” to the damaged spot and the considerable loss of I_2_ to the environment. Finally, when focusing on the Pb coordination environment in the corner-sharing octahedra, the schematic representations within Figures 2 and 4 can also be described by sequential ligand exchange steps that depend on the change in the local chemistry as summarized in Figure S18.

### 2.4 What Compositional Variations Enhance or Attenuate Activated Corrosion Rates in Lead Halide Perovskites?

**Figure 5:**
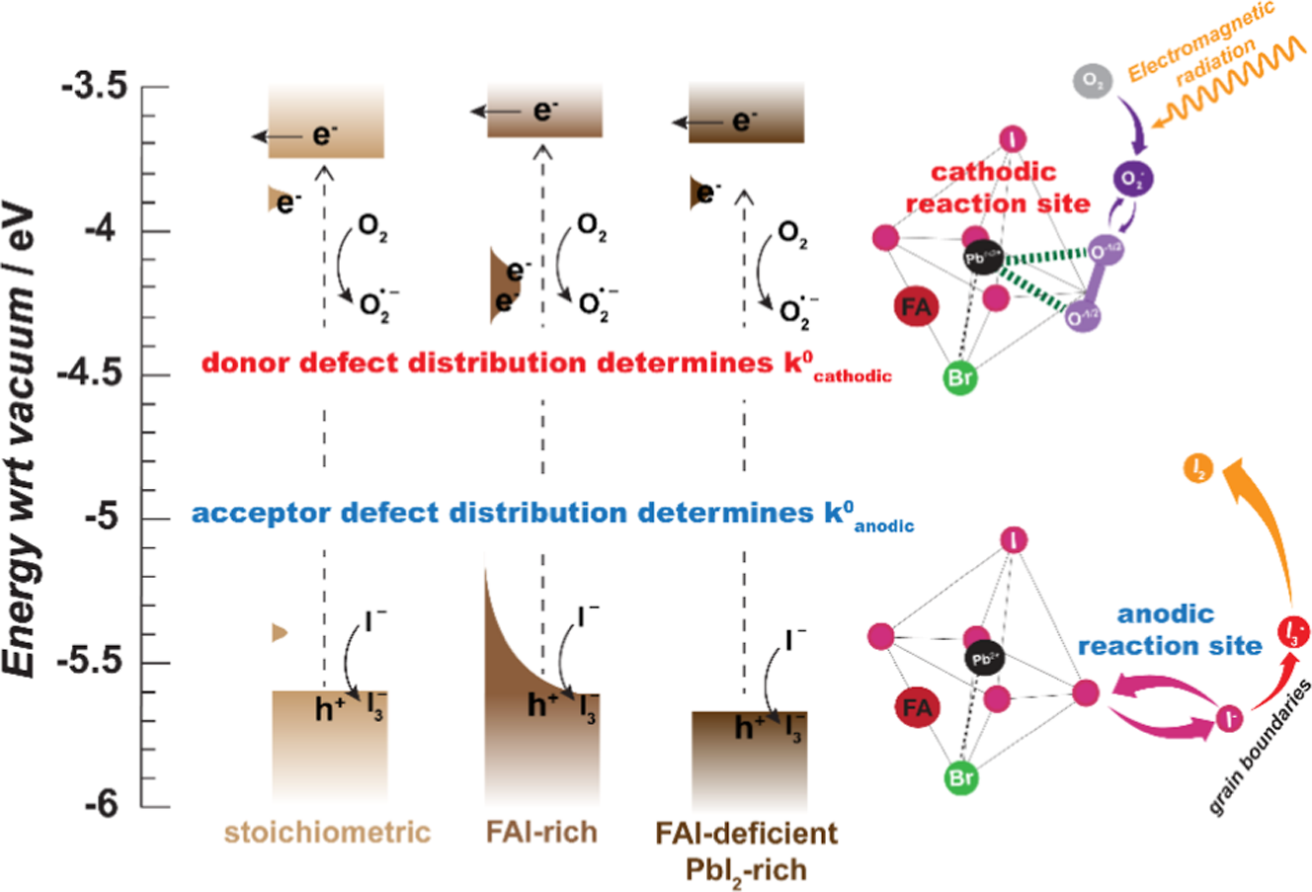
Proposed defect dependence of cathodic and anodic electron transfer of photoexcited electrons in stoichiometric, 2% FAI-rich and 2% FAI-deficient/PbI_2_-rich CsFAMA layers and their influence on the half reactions of the active corrosion mechanism. The energetic distributions of these defect states are derived from our recent specto(electrochemical) studies of these same active layer compositions.^15^ In the FAI-rich material, electrons reside in deeper donor defect levels close to the reduction potential for the reaction of oxygen to superoxide. In the FAI-deficient/PbI_2_-rich material, holes cannot reside in acceptor defect levels so that I_3_^–^ oxidation slows down. Energy distribution of defects adapted from De Keersmaecker et al.^15^ Copyright 2024 American Chemical Society.

Active corrosion processes are generally known to be mitigated by lowering the rates of either (coupled) anodic or cathodic site reactions.^96−98^ To demonstrate this type of degradation mechanism applies to lead halide perovskite materials, we studied CsFAMA films with compositional differences (<2% increases or decreases in FAI precursor concentrations) that we have recently shown significantly affect donor and acceptor defect concentrations and energetic distributions.^15^ For the FA-rich film, (spectro)electrochemical studies show a broad energetic distribution of anion defects distributed from valence band maximum well into the middle of the bandgap due to unreacted iodide anions as well as deep cation defect states (Table 2).^13−15^ XRD data in Figure S1 show that small excesses of FAI precursor lead to perovskite active layers with no detectable PbI_2_ inclusions. These films also exhibit diminished photoluminescence yields and lifetimes, coupled with significantly diminished photovoltaic performance.^15^ To connect these defects to reaction chemistries, we show the NAP-XPS data for such active layers in Figure S19. The FAI-rich system demonstrates a similar pathway, but more rapid time-dependent formation of Pb_WC_ and loss of Pb_SC_ centers versus the stoichiometric active layers (Figures 2B and 4B) with reaction rates shown in Table 2, described in Supporting Information Note 7 and Figure S20. The half-life of the corrosion processes decreases compared to the stoichiometric perovskite, which is expected given the enhanced concentration of acceptor defect states that triggers increased I_3_^–^ formation (Figure 5) and alkali metal iodide formation (Figure S21).^100^ Additionally, the measured deeper donor defects are energetically better aligned with the standard reduction potential of the O_2_/O_2_^•–^ reaction at −0.55 V vs Fc/Fc^+^ (or −4.25 eV vs local vacuum), which supports easier electron transfer to increase O_2_^.-^ formation rates (Figure 5).^101^ The illuminated perovskite material, now extensively depleted of iodides and filled with I_3_^–^ species (Figure S22), first shows bromide-enrichment (Figure 4D) and then a faster restoration (Figure 4E) process compared to the stoichiometric perovskite film due to the abundance of iodide in the surrounding perovskite and the reduction of the nearby formed I_3_^–^ species (Figure S22).

The connection between the energy band diagrams and the reactive defects is further supported when considering the PbI_2_-rich precursor solutions (FAI-deficient by <2%). These films exhibit enhanced concentrations of PbI_2_ inclusions in the active layer (Figure S23—from the analysis of multiple STEM-HAADF images in Figure 3) relative to the stoichiometric perovskites that change degradation parameters as previously demonstrated in perovskite energy conversion and light emitting devices.^102−104^ For the FAI-deficient/PbI_2_-rich perovskite films, NAP-XPS experiments show no Pb_WC_ centers and the immediate formation of Pb_SC_ species, which then slowly attenuate after return to UHV conditions to partly restore the initial PbI_6_-like octahedral environment, as described in Supporting Information Note 8 and Figure S24. Electrochemical studies in literature have shown no detectable anodic defect states, with similar band edges and cation defect distributions as in the stoichiometric perovskite film, as shown in Table 2.^13−15^ In this case, the O_2_ removal does not influence the reactions and rates. Rather, in Figure 5, we posit that the lack of Pb_WC_ is explained by the lack of detectable acceptor defect states (or iodide deficiency; limit of detection is ∼10^13^ defects/cm^3^). A reduced acceptor defect density would arguably decrease the rate of hole transfer that drives the anodic process of the corrosion cell. We would expect a reduction in the formation of I_3_^–^ such that the Pb center atom is immediately surrounded by bromide anions to directly compete with superoxide formation. In summation, the reduction in mobile iodide suppresses the rate of I_3_^–^ generation, which is indeed barely detectable in the high resolution I 3d core level spectra in Figure S25. We hypothesize that the relative ratio of Pb_WC_ centers remains below 0.01, a concentration not detectable by XPS.

Collectively, these three different perovskite formulations discussed above provide a strategic direction for increasing stability in metal halide perovskites for a variety of (opto)-electronic device platforms. We emphasize that corrosion can only be reduced and that for these systems specifically, any modifications to the perovskite chemistries will either increase or decrease relative oxidation and reduction processes. This also provides a powerful opportunity for chemists, in that there is most likely more than one viable pathway to improving materials stability, if functionality can be retained.

## 3 Perspective and Conclusions

Within the metal halide perovskite community, individual studies have generated several proposed degradation pathways without clear consensus, ranging from intrinsic defects, to spontaneous phase segregation to electrochemical reactions or reactions with electrical contacts, as well as electronic and interfacial property changes.^105−107^ The result is a highly material and stressor dependent description of degradation pathways that challenges theoretical and characterization efforts. NAP-XPS has demonstrated to be a powerful tool to the characterization of near-surface O_2_/X-ray/white light induced active corrosion within triple cation mixed halide perovskite films and is expected to be readily extrapolated to other ambient gases. We propose that photogenerated electrons from X-ray and white light excitation, residing close to the CBM, drive superoxide formation while photogenerated holes in the VBM oxidize iodide anions which have been displaced by bound superoxide. These reactions are predicted to occur at electronically coupled but spatially separated (nm-length scale) cathodic sites of weakly coordinated lead/superoxide and iodide-rich anodic sites, accompanied by the creation of a steep electrochemical gradient through the film, a concept that forms the basis for a variety of active corrosion mechanisms. The anodic reaction causes near-surface bromide enrichment in the form of more strongly coordinated bromide-enriched [PbX_6_]^4–^ octahedra, consistent with “de-mixing” proposed previously for mixed halide perovskites under stress. When stress conditions are removed (return to UHV), the surrounding perovskite material and reoxidized I_3_^–^ species can act as a “reservoir” of replacement iodide to feed the repair of the damaged region consistent with previously suggested “self-healing” effects. Even small compositional changes can accelerate the active corrosion process (FAI-rich films), while PbI_2_-rich (free iodide-deficient) stoichiometries change the degradation mechanism, which has been previously inferred in stability studies of perovskite energy conversion and light emitting devices.

Recognizing that chemical characterization in combination with the understanding of the electronics will be essential to design and optimize next-generation optoelectronic platforms, this in situ NAP-XPS characterization approach can be an important asset in the investigation of interface engineering for top contact (and bottom contact) modifications used as mitigation strategies in device optimization, with focus on photoelectrochemical stability, especially when combined with (spectro)electrochemical characterization approaches. We propose that NAP-XPS, in the presence of reactive gases, with and without illumination and temperature excursions can be used more broadly to rapidly characterize interface modifications which have been proposed to provide a paradigm shift in the local control of perovskite degradation chemistry and improvements in long-term device stability, a critical goal in the creation of viable energy conversion platforms.

## Supporting Information Available

The Supporting Information document contains experimental procedures; stressors in experimental protocols; XPS and XRD characterization; photoelectrochemical reaction mechanism; Why no decomposition of organic cations?; The role of adsorbed oxygen; origin of strongly coordinated Pb atoms; gain and loss of strongly coordinated Pb atoms and degradation of FAI-rich and PbI_2_-rich perovskites (PDF)
