# Supplementary material for: Activated Corrosion and Recovery in Lead Mixed-Halide Perovskites Revealed by Dynamic Near-Ambient Pressure X-ray Photoelectron Spectroscopy
Source: J Am Chem Soc. 2025 Feb 27;147(10):8881–92. doi: 10.1021/jacs.5c00668 (PMC11912474; doi:10.1021/jacs.5c00668)
Supplement: Supplementary file 1 — ja5c00668_si_001.pdf [file ja5c00668_si_001.pdf]

Supporting Information:

Activated Corrosion and Recovery in Lead Mixed-Halide Perovskites Revealed by Dynamic Near-Ambient Pressure X-ray Photoelectron Spectroscopy

Michel De Keersmaecker,[†,‡,*] *, Paul Dietrich,*[#] *Mounib Bahri,*[¶] *Nigel D. Browning,*[¶] *Neal R. Armstrong,*[‡] *and Erin L. Ratcliff*[†,‡,^,*]

[†]Department of Materials Science and Engineering, Laboratory for Interface Science of Printable Electronic Materials, Georgia Institute of Technology, 771 Ferst Drive NW, Atlanta, GA, 30332, USA.

[‡]Department of Chemistry and Biochemistry, The University of Arizona, 1306 E. University Way, Tucson, AZ, 85721, USA

[#]SPECS Surface Nano Analysis GmbH, Voltastraße 5, Berlin, 13355, Germany

[¶]Department of Mechanical, Materials and Aerospace Engineering, University of Liverpool, 506 Brodie Tower, Liverpool, L69 3GQ, United Kingdom

[^]School of Chemistry and Biochemistry, Georgia Institute of Technology, 901 Atlantic Drive NW, Atlanta, GA, 30332, USA

Corresponding author

Email: eratcliff8@gatech.edu, mkeersmaecker3@gatech.edu

Contents

S1 Experimental procedures **S3**

S1.1 Sample preparation S3

S1.2 Perovskite film characterization S4

S1.2.1 $\theta/2\theta$ X-ray diffraction (XRD) S4

S1.2.2 Ultraviolet-visible (UV-Vis) spectrophotometry S4

S1.2.3 Near ambient pressure X-ray photoelectron (NAP-XPS) spectroscopy S4

S1.2.4 Conventional X-ray photoelectron (XPS) spectroscopy S5

S1.2.5 Scanning Transmission electron (STEM) microscopy and Focused Ion Beam (FIB) creation of sub-100 nm lamella for imaging S6

S2 Stressors in experimental protocols: A comparison **S7**

S3 XRD characterization of perovskites **S8**

S4 Suppl. Note 1 - Surface composition of all perovskites **S9**

S5 Suppl. Note 2 - Iodide oxidation accompanies Pb_{WC} formation **S14**

S6 Suppl. Note 3 - The role of adsorbed oxygen **S16**

S7 Suppl. Note 4 - Why no decomposition of organic cations? **S20**

S7.1 N 1s high resolution core level spectra under gas exposure S20

S7.2 C 1s high resolution core level spectra under gas exposure S20

S7.3 N 1s and C 1s high resolution core level spectra under UHV after gas exposure . . S21

S8 Suppl. Note 5 - Photoelectrochemical reaction mechanism **S23**

S9 Suppl. Note 6 - Origin of strongly coordinated Pb atoms **S26**

S10 Gain and loss of strongly coordinated Pb atoms **S28**

S11 Suppl. Note 7 - Degradation of FAI-rich CsFAMA perovskites **S33**

S12 Suppl. Note 8 - Degradation of PbI_2-rich/FAI-deficient CsFAMA perovskites **S37**

S1 Experimental procedures

S1.1 Sample preparation

ITO coated glass slides (sheet resistance 9 - 15 Ω $\square^{-1}$, Colorado Concept Coatings LLC, 96041) were cut into squares (10 x 10 x 1.09 mm^3). All these ITO substrates were prerinsed and sonicated in solutions of sodium dodecyl sulfate-water, acetone (ACS, 99.5%, Beantown Chemical), and finally isopropanol (ACS, 99.5%, Beantown Chemical), and allowed to dry. $(Cs_{.05}FA_{.79}MA_{.16})Pb(I_{.87}Br_{.13})_3$ films (or later called $Cs_{.05}FA_{.79}MA_{.16}$) are deposited starting from a precursor solution containing ca. 110 mg of formamidinium iodide (FAI, >99.99%, Greatcell Solar), ca. 324 mg lead (PbI_2, >98%, TCI America), ca. 14 mg of methylammonium bromide (MABr, >99.99%, Greatcell Solar), ca. 47 mg of lead bromide ($PbBr_2$, >98%, TCI America), and ca. 11 mg of cesium iodide (CsI, 99.99% trace metals basis, Acros Organics). For the PbI_2-rich (or $Cs_{.05}FA_{.75}MA_{.16}$) films, the precursor solution contains ca. 90 mg of FAI, ca. 324 mg PbI_2, ca. 14 mg of MABr, ca. 47 mg of $PbBr_2$, and ca. 11 mg of CsI. For the FAI-rich (or $Cs_{.05}FA_{.83}MA_{.16}$) films, the precursor solution contains ca. 119 mg of FAI, ca. 324 mg PbI_2, ca. 14 mg of MABr, ca. 47 mg of $PbBr_2$, and ca. 11 mg of CsI. All these salts were transferred to a hot 2-dram vial (stored at 150°C to minimize water content) in air with a micro stir bar and then immediately loaded into the nitrogen glovebox. Within the glovebox, 512 μL of N,N-dimethylformamide (DMF, anhydrous, 99.9%, Sigma Aldrich) and 128 μL of dimethylsulfoxide (DMSO, 99+%, Alfa Aesar) were added to the vial (generating solution concentrations of ca. 1:1.1:0.2:0.2:0.05 M for FAI:PbI_2:MABr:$PbBr_2$:CsI, respectively) and then the precursor solution was stirred for 20 minutes. The solution was then filtered with 0.22 μm PTFE syringe filter (VWR). Before film processing, the circulation in the glovebox was turned off. The precursor solution was dispensed onto the cleaned ITO substrates (± 30 μL) at room temperature and then spin-cast at 1000 RPM for 10 seconds (1000 acceleration) followed immediately by 4000 RPM for 35 seconds (6000 acceleration). With 10 seconds remaining on the spin-coating cycle, 100 μL of chlorobenzene (anti-solvent, extra dry, 99.8%, Acros Organics) was added to the film in a clean dispensing motion as close to the center/top-surface of the spinning film as possible. All films were immediately transferred to a hot plate for 40 minutes pre-heated at 100°C. The glovebox was purged slightly during film processing.

S1.2 Perovskite film characterization

S1.2.1 $\theta/2\theta$ X-ray diffraction (XRD)

For $\theta/2\theta$-XRD experiments, perovskite films on ITO were mounted on a clay holder in air and loaded into a Phillips X'PERT MPD system, with "PreFIX" source module and a "X'Celerator" detector module, using CuK_α radiation (λ_{max} = 1.541874 Å) with electron gun cathode-anode power settings of 45 kV at 40 mA). Films were analyzed from 5° to 90° in 2θ with 0.0167° step size, scanning symmetrically (i.e., source and detector at same angle with respect to the surface normal thus preferentially detecting scattering from crystal planes parallel to the substrate plane) with a total scan time of approximately 35 minutes. Optical hardware settings include a 0.04 rad soller slit (source and detector side), a 2° divergence slit at 140 mm from sample (source side), a 11.6 mm horizontal mask (source side), a 0.02 mm-thick Nickel filter (source side), and an overhead sample beam knife. Processing/Analysis of XRD patterns were performed with X'Pert HighScore (for background subtraction) and Mercury 2020.3.0 software (for identifying Bragg peaks from crystal structure).

S1.2.2 Ultraviolet-visible (UV-Vis) spectrophotometry

Absorbance data of the perovskite films were measured using an Ocean Optics Balanced Deuterium Tungsten Source (210-2500 nm) and an OCEAN-FX-XR1 fiber optic spectrometer (200-1025 nm) with a 25 μm slit controlled with the Oceanview software package. The perovskite film spectra were collected relative to a cleaned bare ITO substrate.

S1.2.3 Near ambient pressure X-ray photoelectron (NAP-XPS) spectroscopy

An EnviroESCA (SPECS Surface Nano Analysis GmbH, Germany) NAP-XPS system is used to acquire spectra by scanning the kinetic energy across a predefined range (Fixed Analyzer Transmission Scan Mode) at a photoelectron emission angle of 0° and a source-to-analyzer angle of 55° using a small area analyzer lens. The source is based on monochromatic Al K_α excitation at 1486.7 eV with a power of 42 W with a spot size of 0.25 x 0.25 mm^2 with photoemission collection through a 300 μm fixed aperture and 60 mm iris diameter within the lens column in the spectrometer. In the measurement chamber of the EnviroESCA, the sample is illuminated using a Schott KL 2500 LED source with high power white LEDs with a luminuous flux of 1100 lm, which includes UV-vis illumination relevant for perovskite photovoltaics. The dwell time for all data accumulation is 0.2 s. The bias voltage is set at 200 V and the detector voltage at 1530 V. The pass energy

for the survey spectra is set at 100 eV (2 scans), while the analyzer pass energy is changed to 30 eV for the high-resolution spectra (10 scans). The total measurement time for each survey is approximately 5 minutes and for each scan of the high-resolution Pb 4f and I 3d spectra is 6 minutes. The step size for all XPS measurements is set for 0.1 eV. The binding energy is corrected for surface charging with respect to the I 3d component measured for the Pb-I_6 octahedron at 619.1 eV. Curve fitting of core-level spectra was done using CasaXPS fitting software (Casa Software Ltd) using a 70% Gaussian/30% Lorentzian product function peak shape model in combination with a Shirley background to remove the contributions from inelastically scattered photoelectrons. Generally, the full width at half maximum (FWHM) was set as a free parameter but constrained to be the same for all peaks within the same core-level spectrum and was cross-checked against FWHM values needed to describe lineshapes in both PbI_2 and $PbBr_2$ standard samples. The regions are individually defined, and the spectra were fitted with a minimum set of peak components using constraints based on known spin orbital splitting between XPS peaks of the same element. Elemental ratios were calculated using the peak areas fitted using the CasaXPS software and corrected with the relative sensitivity factors (RSFs) normalized to the sensitivity of C 1s for the EnviroESCA system, which are calculated based on the cross section of the excitation energy, the angular asymmetry of photoemission, and the inelastic mean free path (IMFP) for the kinetic energy of the peak center.

S1.2.4 Conventional X-ray photoelectron (XPS) spectroscopy

XPS (monochromatic Al K_α excitation at 1486.3 eV, 10 mA, 15 kV, pass energy of 20 eV) spectra are acquired at a photoelectron take-off angle of 0° (normal to the surface) using a Kratos Axis Ultra PES system (Kratos Analytical, USA) in ultra-high vacuum with a base pressure around 2 x 10^{-9} Torr. All samples are fixed using carbon tape and grounded to stainless steel stubs. The perovskite samples are introduced in the spectrometer through an argon-filled glovebox. The XPS binding energy scale is calibrated with sputter-cleaned Cu, Au, and Ag foils. The XPS spectra are processed using the CasaXPS software package (Casa Software Ltd). The raw XPS data undergo a Shirley background correction and all remaining core level peaks are fit to known chemical components using a 70% Gaussian/30% Lorentzian line shape. To minimize random errors, relative peak shape, width and shifts are held constant, which is extremely important when multiple species are used to fit a single peak.

S1.2.5 Scanning Transmission electron (STEM) microscopy and Focused Ion Beam (FIB) creation of sub-100 nm lamella for imaging

All cross-section STEM specimens preparation were carried out using a dual beam Focused Ion Beam (FIB, Thermofisher Helios 600i).[1] A carbon coated 10 nm layer were first deposited using a carbon high vacuum sputter at a pressure of 5 x 10^{-5} mbar followed by C and Pt deposition of 1 μm each using the FIB Ion-Beam. After etching the surrounding protected area at 30 kV and 9.3 nA, the lamella was lifted out and attached to a TEM grid. The thinning process was carried out first at 16 kV, 50 pA for a thickness down to 2 or even 1 μm. Below 1 μm and down to 500 nm, 8 kV and 21 pA was used. From 500 nm down to less than 100 nm, a 5 kV and 15 pA ion beam was used. A 5 minute final polishing step was used at 2 kV and 5 pA to remove the surface amorphous layer. These multiple low kV steps and the concurrent thinning process are used to minimize ion beam damage, which is critical to preserve the crystalline features within the perovskite sample. All samples in this work have been prepared under the same conditions.

High angle annular dark field (HAADF) scanning transmission electron microscopy (STEM) imaging was performed using a Cs-corrected TEM/STEM JEOL 2100F Cs microscope operating at 200 kV. To make sure the perovskite structure is not damaged, it is critical to use a low electron dose rate of 25 e/$Å^2$s for all the images in this work. Elastically scattered electrons transmitted through the thin sample are captured using a HAADF detector with an angular range between 61 and 163 mrad and a pixel size of 2048 x 2048. For the acquisition of the STEM image, a dwell time of 6 μs was used.

S2 Stressors in experimental protocols: A comparison

Table S1 Differences between earlier (NAP-)XPS studies and what is different in our study

reference	perovskite stack	beam energy	experimental time	aging conditions
Chem. Mater. 2015, 27, 1720-1731[2]	$MAPbI_3$-xCl_x/TiO_2/FTO	4000 eV	2h corrosion before experiment; in some cases, samples were heated for 20 mins; no idea how long samples are exposed to X-rays	single drop of DI water on tilted $MAPbI_3$/TiO_2/FTO and then allowing it to dry in air for 2 h; color change was observed; time evolution was measured under argon and ambient air
ACS Appl. Mater. Interfaces 2020, 12, 43705[3]	$MAPbI_3$/FTO	1486.6 eV	30 mins heating; 5 mins stabilization; 10 mins X-ray exposure	MilliQ water was introduced through a leak valve and then freeze degassed; 5 mbar H_2O; films were heated to target temperature
ChemSusChem 2020, 13, 5722[4]	$MAPbI_3$/FTO	520 eV; 9.9×10^{11} pho/s (4 sun); 7.4×10^{11} pho/s (3 sun)	60 mins constant exposure to X-rays (4.5 min intervals)	1 mbar H_2O/water vapor; decomposition peaks noticed after 25 and 45 mins depending on the photon flux
ChemSusChem 2020, 13, 5722[4]	$MAPbI_3$/FTO	1486.6 eV; 6.1×10^9 pho/s (0.01 sun)	no information on the X-ray exposure time	no metallic Pb during exposure; no peak shifts or appearance of new components observed; decrease of C 1s and N 1s peak of MA
ACS Nano 2023, 17, 25679-25688[5]	cleaved single crystal $MAPbI_3$	no beam flux and energy	no information on the X-ray exposure time	various water vapor pressures; metallic lead is measured; beam damage after 24 mins based on C 1s spectra
ACS Energy Lett. 2016, 1, 360[6]	$MAPbI_3$/TiO_2 /FTO	1.4×10^{12} pho/s	5 min exposure repeated of the course of 48 hours	peak area loss in N 1s and C 1s; time constant of drop in MA component of N 1s peak is 9.8 h
Nat. Commun. 2017, 8, 15218[7]	$MAPbI_3$/glass	no information on the X-ray exposure time	4 hours aging period	UV/vis and SEM in combination with calculations; exposure to dry air; exposure to tungsten lamp with UV-blocking filter
Small Methods 2023, 7 (11), 2300458[8]	$Cs_{.05}MA_{.15}FA_{.8}$ $Pb(I_{.85}Br_{.15})_3$/ TiO_2/FTO	1486.6 eV; 5.0×10^{13} pho/s	520 mins of exposure	no clear shifts before 200 mins of exposure; after 200 mins of exposure all core levels shift toward lower BEs
Chem. Mater. 2017, 29, 8478[9]	$MAPbI_3$/Au	1486.6 eV	exposed to aging conditions for 6h; length of exposure to X-rays is not entirely clear	temperature was varied from 25 to 300 °C under $\approx 10^{-8}$ mbar; a rate of 5 °C/min; 15 mins stabilization; under 1 mbar pressure of O_2 or H_2O
this manuscript	$Cs_{.05}FA_{.79}MA_{.16}$ /ITO; $Cs_{.05}FA_{.83}MA_{.16}$ /ITO; $Cs_{.05}FA_{.75}MA_{.16}$ /ITO	1486.6 eV; 1.7×10^{11} pho/s	18 min total X-ray exposure time per sample per element; 6 min exposure for the other spot per element	mainly surface corrosion; white LED light; no heating; no water exposure; no ambient air exposure; no color change was observed

S3 XRD characterization of perovskites

In Figure S1, the stoichiometric composition, $Cs_{.05}FA_{.75}MA_{.16}$, reveals small concentrations of excess PbI_2, which have previously suggested to decrease nonradiative recombination rates and increase carrier lifetimes,[10] while the nonstoichiometric perovskite films either present higher PbI_2 concentrations, in the case of PbI_2-rich $Cs_{.05}FA_{.79}MA_{.16}$, or no excess PbI_2, in the case of FAI-rich $Cs_{.05}FA_{.83}MA_{.16}$, strongly suggesting that unreacted FA^+ and iodide ions are present in both cases.

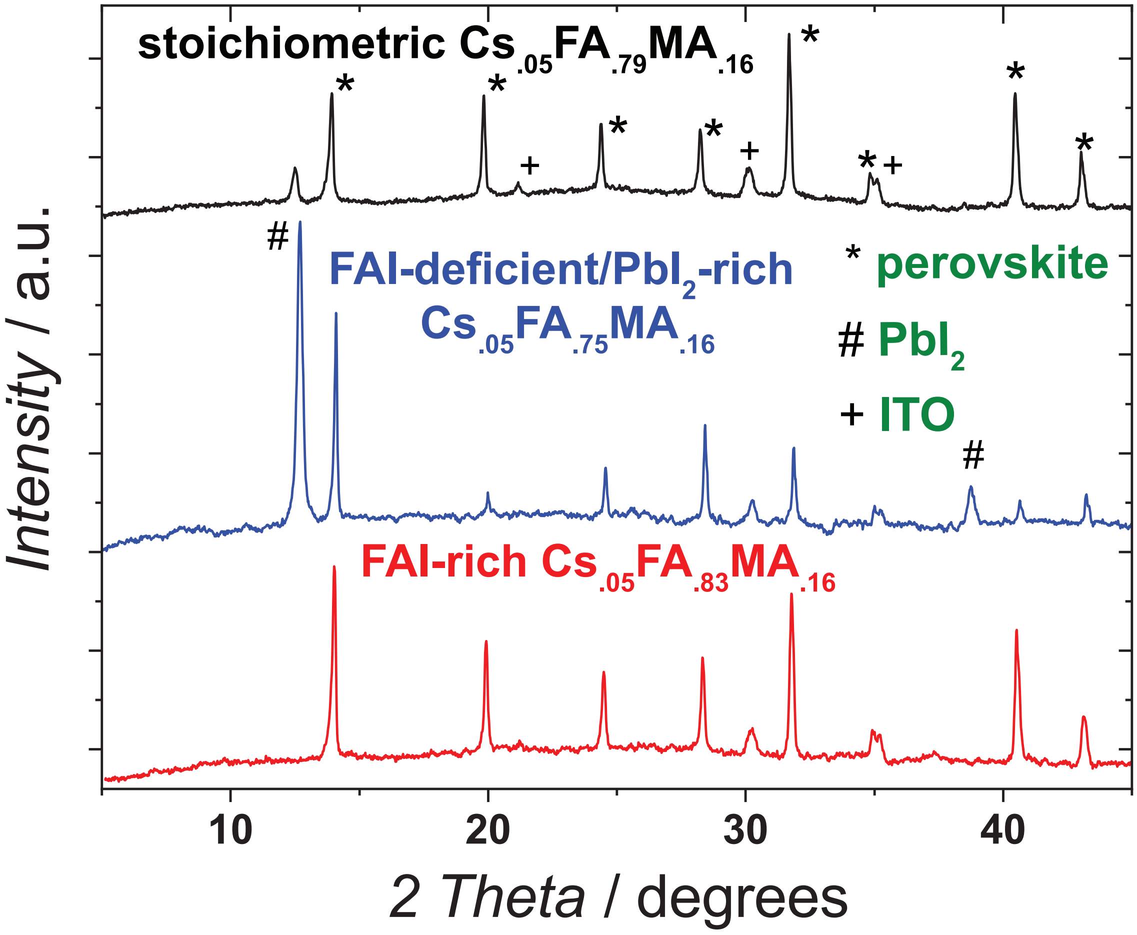

Figure S1 XRD spectra of stoichiometric $Cs_{.05}FA_{.79}MA_{.16}$, and PbI_2-rich/FAI-deficient $Cs_{.05}FA_{.75}MA_{.16}$ and FAI-rich $Cs_{.05}FA_{.83}MA_{.16}$ films on ITO/glass substrates.

S4 Suppl. Note 1 - Surface composition of all perovskites

(Electro)chemical interfacial interactions at perovskite thin films under chosen atmospheric conditions (in this case, dry O_2) can be systematically studied using near-ambient pressure X-ray photoelectron spectroscopy (NAP-XPS) based on a specific experimental procedure described in Figure 1. First, the perovskite sample is exposed to X-rays at normal fluence for XPS and its composition is measured and verified under vacuum. Then, 2 mbar of dry O_2 is added to the measurement chamber in the presence of X-ray and white light, without change in sample position, so that the same data can be collected in the same region under O_2 gas exposure. After all spectra are acquired the measurement chamber is evacuated so that a new data set under vacuum is collected, again in the same sample region. Finally, a new spot is measured, away from the original analysis region, to assess whether O_2 exposure alone could have caused compositional changes.

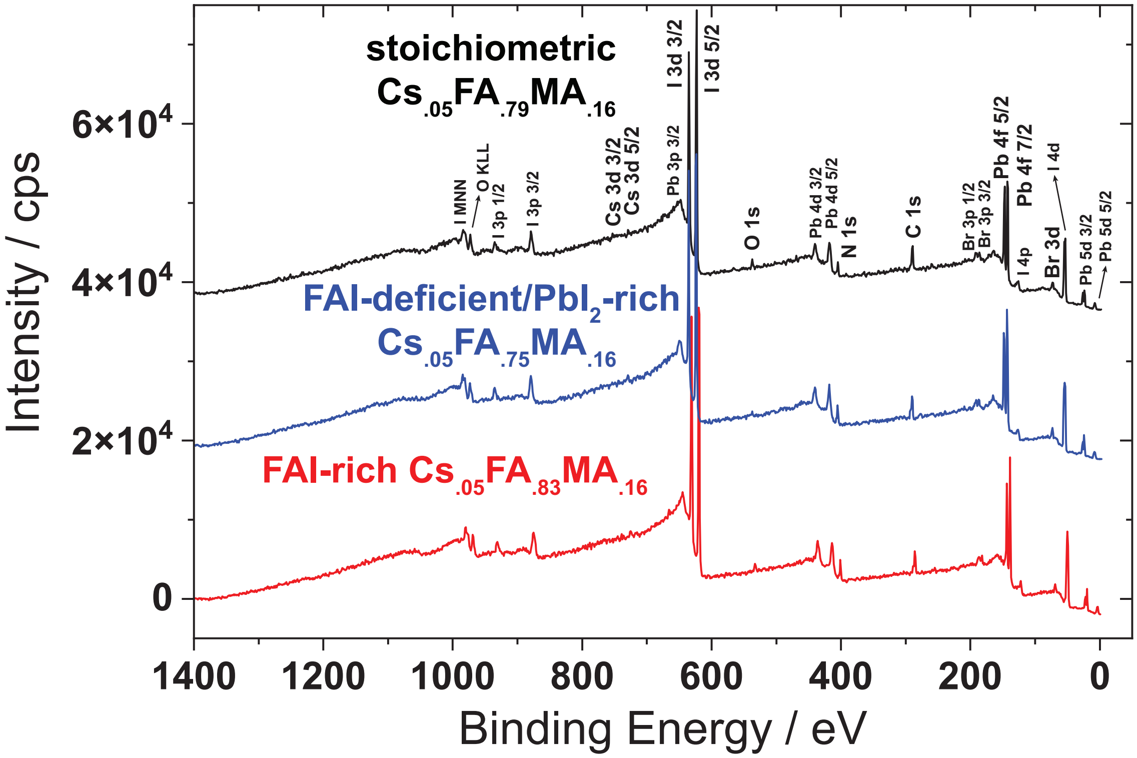

Figure S2 Survey scans of the $Cs_{.05}FA_{.79}MA_{.16}$, PbI_2-rich/FAI-deficient $Cs_{.05}FA_{.75}MA_{.16}$ and FAI-rich $Cs_{.05}FA_{.83}MA_{.16}$ films on ITO/glass substrates under UHV.

First, we focus on the stoichiometric triple cation perovskite films, where we know maintaining stoichiometry during precursor preparation for manufacturing is extremely important because small deviations going from a deficit of FAI (PbI_2-rich) to an excess of FAI (FAI-rich) have demonstrated specific benefits and disadvantages for the performance of perovskite solar cells,[11] while XRD spec-

tra have shown minimal differences as demonstrated in Figure S1. Similarly, compositional differences do not significantly impact the optical band gap as seen in a previous communication of our group.[12] The elemental composition is measured using survey scans in Figure S2 with the EnviroESCA system under ultra-high vacuum conditions to verify no impurities are present in all three perovskite films. The relevant experimental elemental ratios including Pb/I, Pb/Cs and Pb/Br are calculated based on the Pb 4f, I 3d, Cs 3d, Br 3d, O 1s, N 1s, C 1s high-resolution core level spectra as demonstrated in Table S2 by considering the relative sensitivity factors (RSFs) using Scofield photoionization cross-sections of the excitation energy (1486.7 eV),[13] the IMFP for the kinetic energy of the peak center,[14] and the angular asymmetry of photoemission as described in Table S2. Under high pressures, quantification needs to consider the loss of inelastically scattered photoelectrons due to collisions with gas molecules that are present close to the surface as explained in Figure S3.[15–17] From control experiments on an ionic liquid, C_3C_1im NTf_2, quantification results (relative atomic ratios) are only different by a few percent versus those ratios acquired in high vacuum and the ratios expected from the chemical composition of the ionic liquid for background pressures near $\sim$ 2 mbar.[18] For our experiments, this means we can use the RSFs based on the Scofield photoionization cross-sections and corrected for the instrument parameters as shown in Table S3 for both the quantifications under UHV and NAP-XPS.

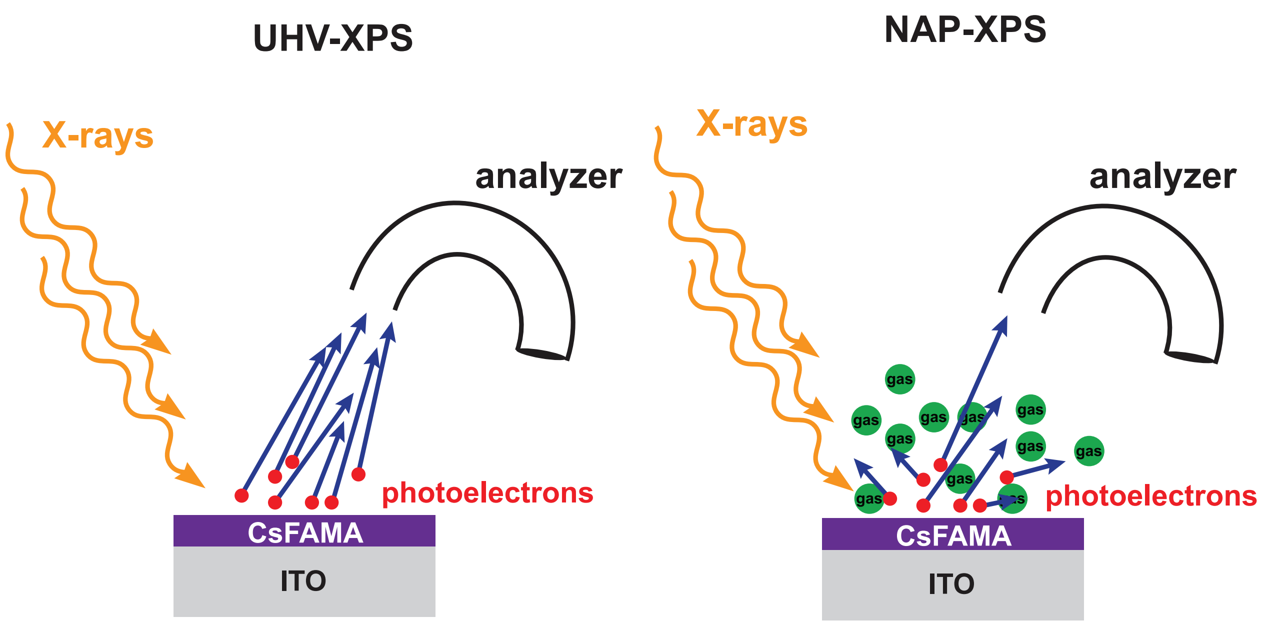

Figure S3 Schematic of the UHV-XPS versus the NAP-XPS experiment, which explains the difference in relative sensitivity factor.

These experimental ratios are then directly compared to the expected composition ratios starting

Table S2 Calculated (or expected composition from precursor solution) and measured relative atomic ratios using the RSFs from the high-resolution Pb 4f, I 3d, Cs 3d, Br 3d, N 1s and C 1s core level spectra for the $Cs_{.05}FA_{.79}MA_{.16}$, PbI_2-rich $Cs_{.05}FA_{.75}MA_{.16}$ and FAI-rich $Cs_{.05}FA_{.83}MA_{.16}$ films. The experimental ratios are from the first and the second spot.

Ratio	stoichiometric $Cs_{.05}FA_{.79}MA_{.16}$ (calculated)	stoichiometric $Cs_{.05}FA_{.79}MA_{.16}$ (experimental spot 1)	stoichiometric $Cs_{.05}FA_{.79}MA_{.16}$ (experimental spot 2)
I/Pb	2.61	3.8[1]	3.7[1]
Cs/Pb	0.05	0.1	0.1
Br/Pb	0.39	0.4	0.3
N/Pb	1.74	2.2	2.2
FA(N)/Pb	1.58	1.9	1.9
MA(N)/Pb	0.16	0.3	0.3
C/Pb	0.95	2.4	2.1
MA(C)/Pb	0.16	1.2	1.0
FA(C)/Pb	0.79	1.2	1.1
O/Pb	-	1.5	1.5
Ratio	FAI-deficient/PbI_2-rich $Cs_{.05}FA_{.75}MA_{.16}$ (calculated)	FAI-deficient/PbI_2-rich $Cs_{.05}FA_{.75}MA_{.16}$ (experimental spot 1)	FAI-deficient/PbI_2-rich $Cs_{.05}FA_{.75}MA_{.16}$ (experimental spot 2)
I/Pb	2.55	3.8[1]	3.7[1]
Cs/Pb	0.05	0.1	0.1
Br/Pb	0.39	0.4	0.3
N/Pb	1.66	2.2	1.9
FA(N)/Pb	1.5	1.9	1.7
MA(N)/Pb	0.16	0.3	0.2
C/Pb	0.91	2.7	2.5
MA(C)/Pb	0.16	1.3	1.4
FA(C)/Pb	0.75	1.3	1.1
O/Pb	-	0.6	1.0
Ratio	FAI-rich $Cs_{.05}FA_{.83}MA_{.16}$ (calculated)	FAI-rich $Cs_{.05}FA_{.83}MA_{.16}$ (experimental spot 1)	FAI-rich $Cs_{.05}FA_{.83}MA_{.16}$ (experimental spot 2)
I/Pb	2.73	4.5[1]	3.7[1]
Cs/Pb	0.05	0.1	0.1
Br/Pb	0.39	0.4	0.4
N/Pb	1.82	2.4	2.5
FA(N)/Pb	1.66	2.2	1.2
MA(N)/Pb	0.16	0.2	0.3
C/Pb	0.99	2.6	2.6
MA(C)/Pb	0.16	1.1	1.2
FA(C)/Pb	0.83	1.5	1.4
O/Pb	-	0.9	1.1

[1]increased I/Pb ratio due the iodide surface enrichment and increased amount of FAI

Table S3 Relative sensitivity factors (RSFs) starting from the Scofield photoionization cross-sections based on the cross section of the excitation energy (1486.7 eV),[13] the angular asymmetry of photoemission, and the IMFP for the kinetic energy of the peak center[14] within the EnviroESCA system.

Core level electron	Scofield RSF for EnviroESCA
Pb 4f	24.67
I 3d	26.41
Cs 3d	28.70
Br 3d	3.20
N 1s	1.67
C 1s	1.00
O 1s	2.47

Table S4 Calculated (based on the expected composition) and measured relative atomic ratios from Pb 4f, I 3d, Cs 3d, Br 3d, N 1s and C 1s core level spectra for $Cs_{.05}FA_{.79}MA_{.16}$, $Cs_{.05}FA_{.83}MA_{.16}$ (FAI-rich) and $Cs_{.05}FA_{.75}MA_{.16}$ (PbI_2-rich) films exposed to 2 mbar of dry O_2 gas. The ratios are calculated based on the total peak area per core level in the average scan, which means we do not consider oxidation state changes (i.e., binding energy shifts) of the species during the measurement.

Ratio	stoichiometric (calc)	stoichiometric (exp)	FAI-rich (calc)	FAI-rich (exp)	FAI-deficient/ PbI_2-rich (calc)	FAI-deficient/ PbI_2-rich (exp)
I/Pb	2.61	2.6	2.7	3.1[1]	2.55	2.9
Cs/Pb	0.05	-[2]	0.05	-[2]	0.05	-[2]
Br/Pb	0.39	0.4	0.39	0.4	0.39	0.4
N/Pb	1.74	1.7	1.82	2.1[1]	1.66	1.9
FA(N)/Pb	1.58	1.5	1.66	1.8	1.5	1.4
MA(N)/Pb	0.16	0.3	0.16	0.3	0.16	0.5
C/Pb	0.95	2.4	0.99	3.0	0.91	2.3
MA(C)/Pb	0.16	1.2	0.16	1.1	0.16	1.3
FA(C)/Pb	0.79	0.9	0.83	1.5	0.75	1.0
O/Pb	-	2.2	-	2.1	-	1.8

[1] slightly increased I/Pb and N/Pb ratio due the increased amount of FAI in the precursor solution
[2] not measurable due to the drop in S/N because of the presence of O_2 gas

from the prepared precursor solutions. The trends for the Cs/Pb, Br/Pb, and N/Pb elemental ratios (green in Table S2), as well as elevated I/Pb ratios (orange in Table S2) follow the expected film compositions based on the stoichiometric $Cs_{.05}FA_{.79}MA_{.16}$, PbI_2-rich $Cs_{.05}FA_{.75}MA_{.16}$ and FAI-rich $Cs_{.05}FA_{.83}MA_{.16}$ precursor solutions. The high C/Pb ratios (red in Table S2) are explained by the presence of adventitious carbon and other carbon compounds absorbed to the perovskite surface. Additionally, we have added the O/Pb ratio (blue in Table S2) which seems to be slightly dependent on the perovskite composition. We note that the samples were shipped under an inert atmosphere from UArizona to SPECS in Berlin to keep ambient air out to the best of our abilities.

By exposing the first spot of all three perovskite samples to 2 mbar of dry O_2, similarly all elemental

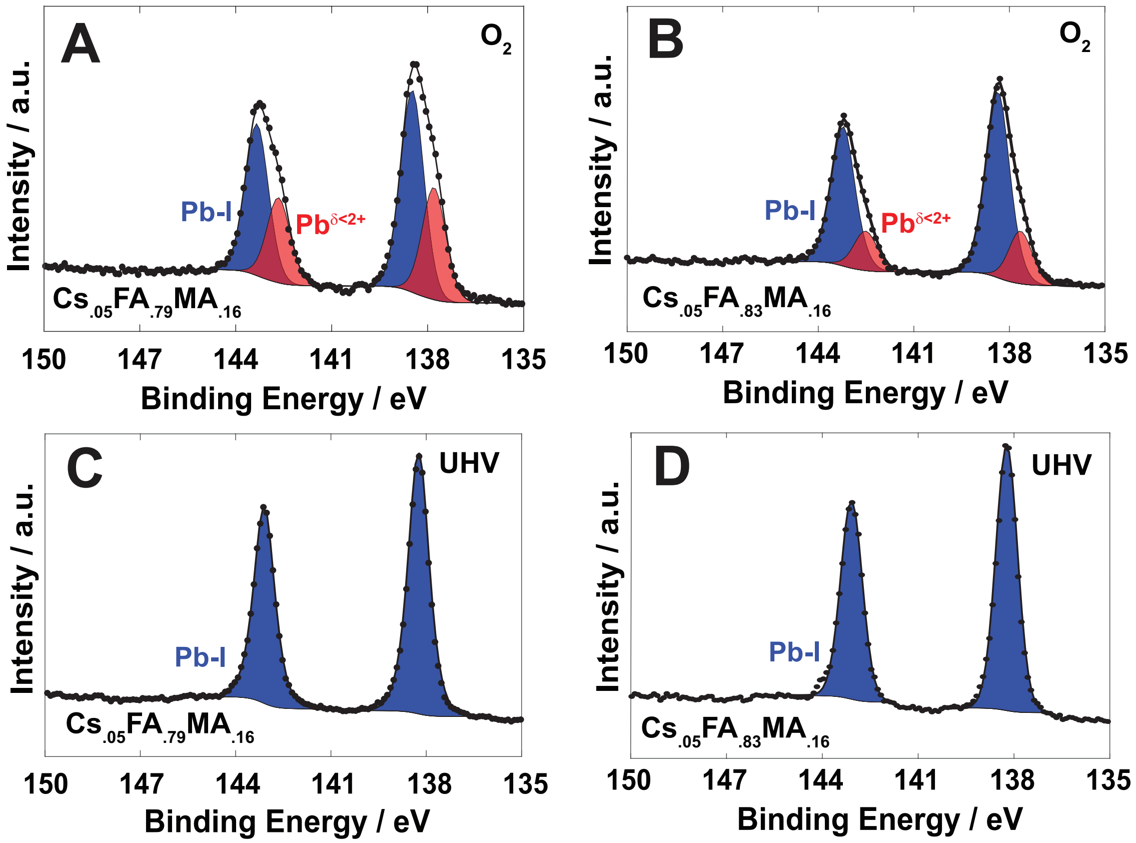

Figure S4 Pb 4f high-resolution XPS core level spectra of a (A) stoichiometric $Cs_{.05}FA_{.79}MA_{.16}$ and (B) FAI-rich $Cs_{.05}FA_{.83}MA_{.16}$ film that demonstrates the presence of a low binding energy shoulder in the Pb $4f_{7/2}$ and Pb $4f_{5/2}$ peaks under dry O_2 gas exposure in the measuring chamber of the XPS instrument. Pb 4f high-resolution XPS core level spectra of a (C) stoichiometric $Cs_{.05}FA_{.79}MA_{.16}$ and (D) FAI-rich $Cs_{.05}FA_{.83}MA_{.16}$ film under UHV.

ratios, except C/Pb (due to adventitious carbon) follow the expected composition as demonstrated in Table S4 using the same color coding. For the O/Pb ratio, we note an increase due to an increase in adsorbed O_2 to the perovskite surface as a result of exposing the perovskite to dry O_2 gas as well as white and X-ray illumination. Another important observation is that only during the exposure of the stoichiometric $Cs_{.05}FA_{.79}MA_{.16}$ and FAI-rich $Cs_{.05}FA_{.83}MA_{.16}$ perovskite surfaces to 2 mbar of dry O_2 gas, a low binding energy (BE) shoulder appears in their respective Pb 4f high-resolution core level spectra as demonstrated in Figure S4A and B. Here, the blue peak at 138.3 eV (also measured in the UHV spectrum in Figure S4C and D) describes the differences between initial and final states of the Pb atom, which are extremely sensitive to differences in the coordination environment of Pb atom replacement of even one I atom within the PbI_6^{4-} octahedron causes small changes in these BEs.[19] The newly formed red peak, with a BE of ca. 137.7 eV, represents a Pb atom with a weaker coordination environment.

S5 Suppl. Note 2 - Iodide oxidation accompanies Pb_{WC} formation

Halide segregation processes under light and bias have been explained based on thermodynamic and kinetic gradients[20] and preferential movement of the most easily oxidized halide.[21] Therefore, we focus iodide anion oxidation because their redox potentials within the halide series ($I^- < Br^- < Cl^-$) explain their domination over bromide and chloride anion oxidation.[21] The high-resolution I $3d_{5/2}$ core level spectra in Figure S5A during O_2 exposure (step II) confirm the growth of a higher BE shoulder at 619.8 eV after ± 5.6 minutes of exposure (i.e., 7 scans), which has been associated with a covalent bond in surface-adsorbed homoatomic molecules of I_2 gas formed following[22]

$$2\,I^- \rightleftharpoons I_2 + 2\,e^- \quad \text{or} \quad 2\,I^- + 2\,h^+ \rightleftharpoons I_2 \tag{S1}$$

where excess I^- can act as a Lewis base and I_2 as a Lewis acid to form triiodide anions, I_3^-.[23]

$$I^- + I_2 \rightleftharpoons I_3^- \tag{S2}$$

The weakly coordinated Pb (or Pb_{WC}) atoms appearing after 3 minutes caused by $O_2^{-\cdot}$ formation is proposed to be triggered by an iodide oxidation process with the formation of photogenerated holes in close proximity to mobile I^-. Photogenerated electron–hole pairs can be separated under local (nm-scale) electric fields, in competition with their immediate recombination, and can subsequently electrochemically react, resulting in adsorbed superoxide ($O_2^{-\cdot}$) at cathodic sites, while the iodide oxidation occurs at nearby (coupled) anodic sites. Spatially heterogeneous environments with local potential gradients across nm length scales provide the electrochemical driving force to enhance ionic movement to the respective reactive sites, feeding this active corrosion process.[24]

From the time evolution of the I_3^- (or chemisorbed I_2) species in Figure S5A, we notice a longer induction period compared to the formation of the Pb_{WC} atoms. This lag is explained by the need for two holes and excess iodide to anodically generate I_3^-, while the cathodic process with the formation of superoxide and weakly coordinated lead only needs one electron. Additionally, the generation of I_3^- quickly saturates in Figure S5A due to the loss of adsorbed I_2 gas under low pressure. Following Le Châteliers principle, this I_2 loss shifts the equilibrium of the surface-specific active corrosion reaction to the right, which explains the boost in Pb_{WC} atom formation. The Br 3d spectra for this perovskite sample in Figure S5B do not show the formation of new Br species, however, the Br/I relative atomic ratios do increase with increasing X-ray excitation exposure.

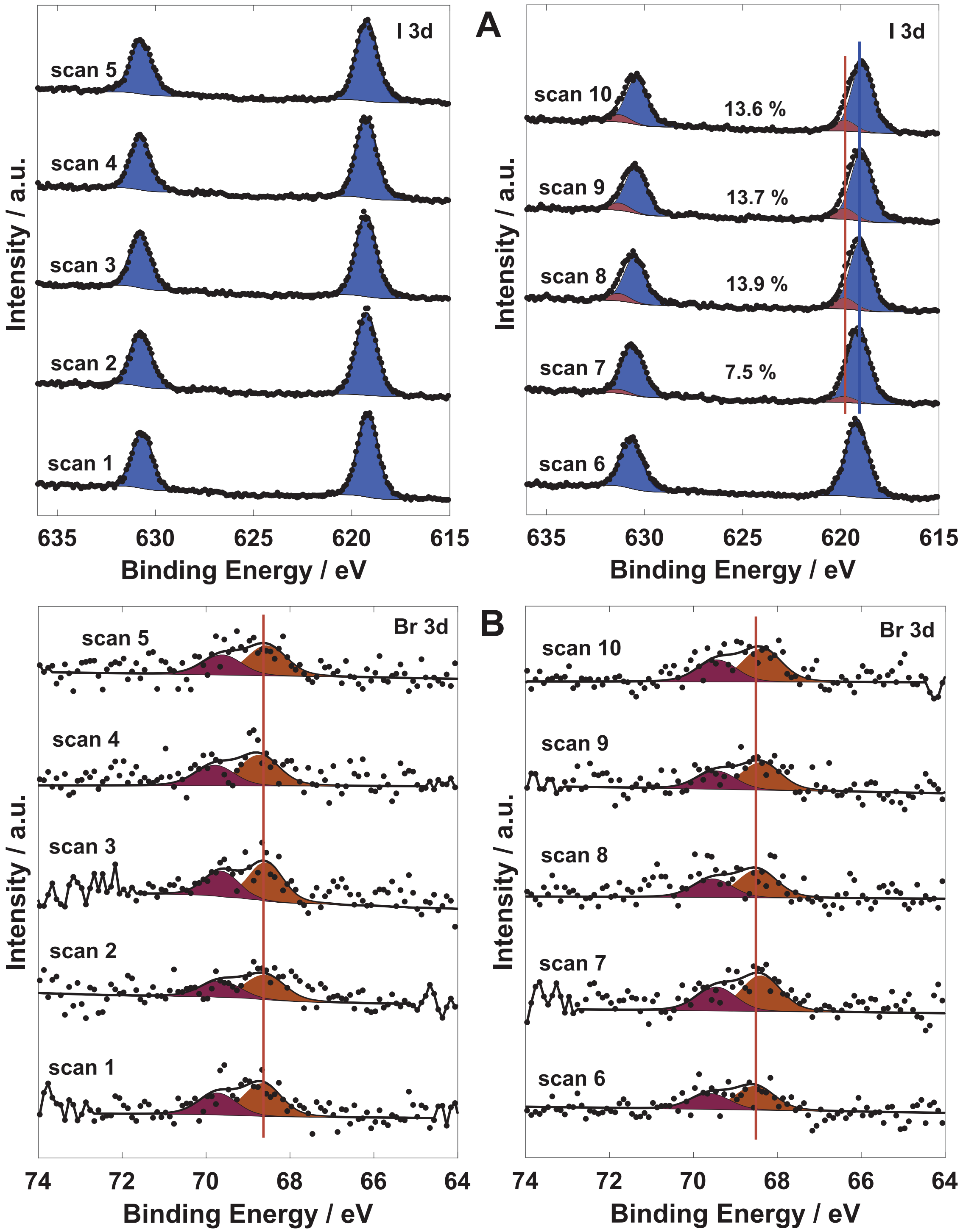

Figure S5 (A) XPS data for the I $3d_{3/2,5/2}$ region as a function of exposure time to X-ray excitation in the presence of 2 mbar O_2 (complement to Figure 2B). A new higher BE shoulder at 619.8 eV (versus 619.1 eV in the PbX_6^{4-} octahedra) is observed, at later times than the appearance of Pb_{WC}, after a 35-minute exposure associated with triiodide anions (I_3^-). (B) XPS data for the Br $3d_{3/2,5/2}$ region as a function of exposure time to X-ray excitation in the presence of 2 mbar O_2. No shifts and new peaks are observed.

S6 Suppl. Note 3 - The role of adsorbed oxygen

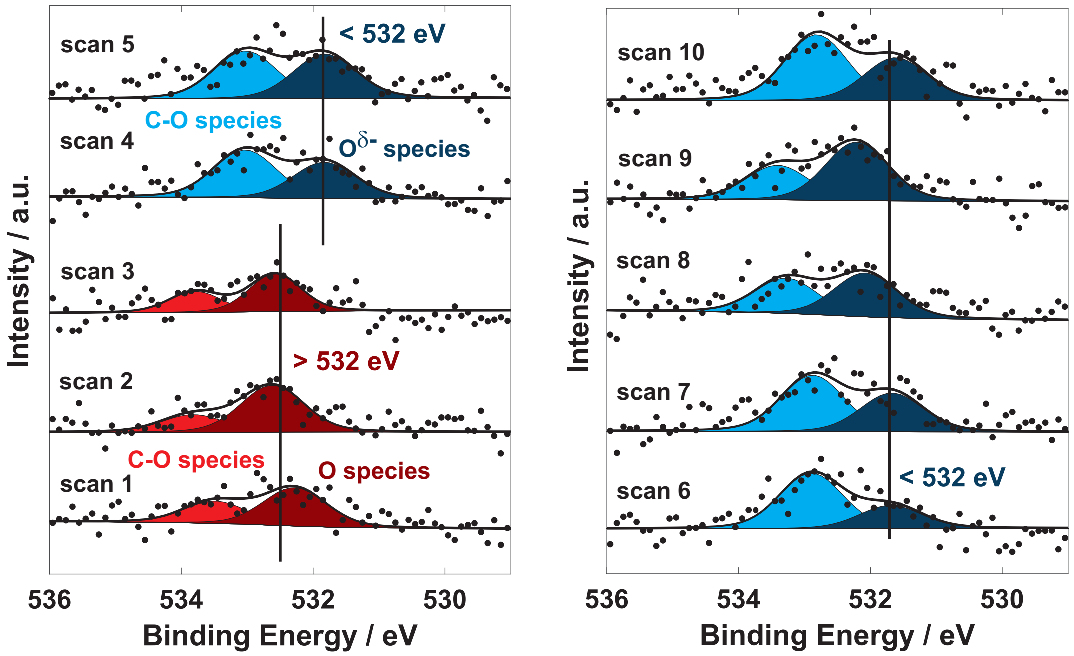

Figure S6 Individual (scan 1 to 10) O 1s high-resolution XPS core level spectra of a stoichiometric $Cs_{.05}FA_{.79}MA_{.16}$ film under dry O_2 gas exposure. The formation of $O_2^{-\cdot}$ and peroxide species explains the BE shift of both the (red) C-O and O species with the formation of more electron-rich (blue) species (i.e., BE decrease) due to reaction of these negatively charged species with the central Pb atom to form Pb_{WC} atoms.

The data in Figure 2B demonstrates that X-rays excite the perovskite from scan 1 to scan 4 without chemical changes. The high-resolution O 1s core level spectra (scan 1 to 4) in Figure S6 corroborate the existence of ionized and weakly adsorbed O_2 species at the charged perovskite surface (i.e., 532 - 533 eV), which are two peaks assigned to weakly adsorbed molecular O_2 and to oxygen bound to carbon (C-O).[25] Based on reports from multiple previous groups,[7,26–28] one must readily acknowledge and accept residual oxygen species in solution processing due to solvent decomposition. Solvent molecules are indeed prone to compete with the iodide ligands to coordinate with Pb^{2+} to form different types of iodoplumbate complexes in solution.[29–31] Other considerations for the origins of oxygen are the oxygen permeability of common encapsulation layers, ambient processing, and the partial pressure of molecular O_2 of 1×10^{-9} Torr even in vacuum. Hence, O_2 gas needs to be considered an important catalyzing factor to capture the reaction chemistries on the time scale and the limit of detection consistent with XPS. To demonstrate this, Figure S7 includes the expanded binding energy region for the O 1s high resolution XPS core level spectra as well as the slowly increasing O 1s elemental concentration within the perovskite sample as a function of

X-ray, white light and O_2 gas exposure time. Additionally, the BE shifts of the O 1s core level peaks for the adsorbed oxygen species are real (i.e. not as a result of surface charging) considering the two narrow peaks of the molecular O_2(g) molecule, which can be used as an internal standard due to its paramagnetic nature defined by 1 eV splitting and an area ratio of 2:1.[32]

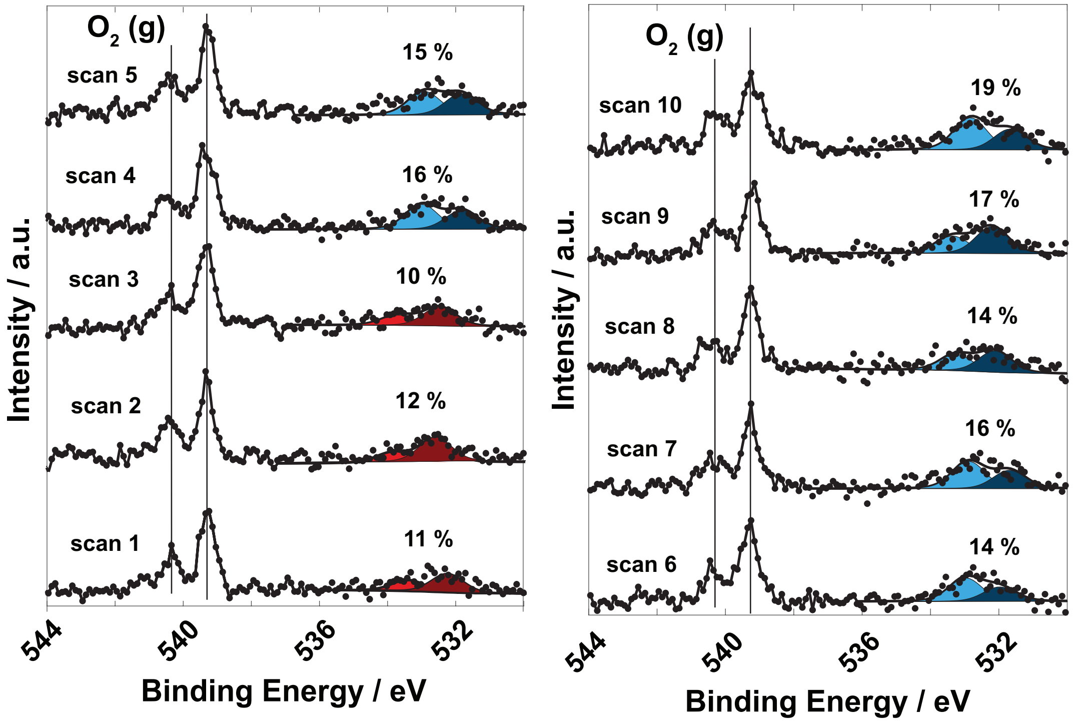

Figure S7 Individual (scan 1 to 10) O 1s high-resolution XPS core level spectra of a stoichiometric $Cs_{.05}FA_{.79}MA_{.16}$ film under dry O_2 gas exposure that includes the O_2 gas binding energy region. The percentage of the O 1s elemental concentration is added for every scan as a function of exposure time.

As a first control, the perovskite film is exposed to X-rays alone under 1×10^{-8} Torr for the same amount of time (I in Figure 1). Here, we measure no significant changes in the Pb 4f and the I 3d core level peak as well as in the O 1s core level peaks in Figure S8. Additionally, no BE shifts are measured and compared to the sample under 2 mbar O_2 pressure, adsorbed oxygen concentrations are lower. As a second control, the perovskite sample is only exposed to 2 mbar O_2 for the same amount of time (IV in Figure 1). Again, we measure no significant changes in the Pb 4f and the I 3d core level peak as well as in the O 1s core level peaks in Figure S9. The envelop that includes the O_2 gas environment on the same counts per second scale masks the O 1s surface species, indicating that if O 1s was sufficient oxidizing agent for any species (PbI_2 or perovskite), there is a very large concentration available to drive corrosion reactions, but they are not observed. Oxygen concentrations have remained similar to the original perovskite sample. Iodide could certainly be

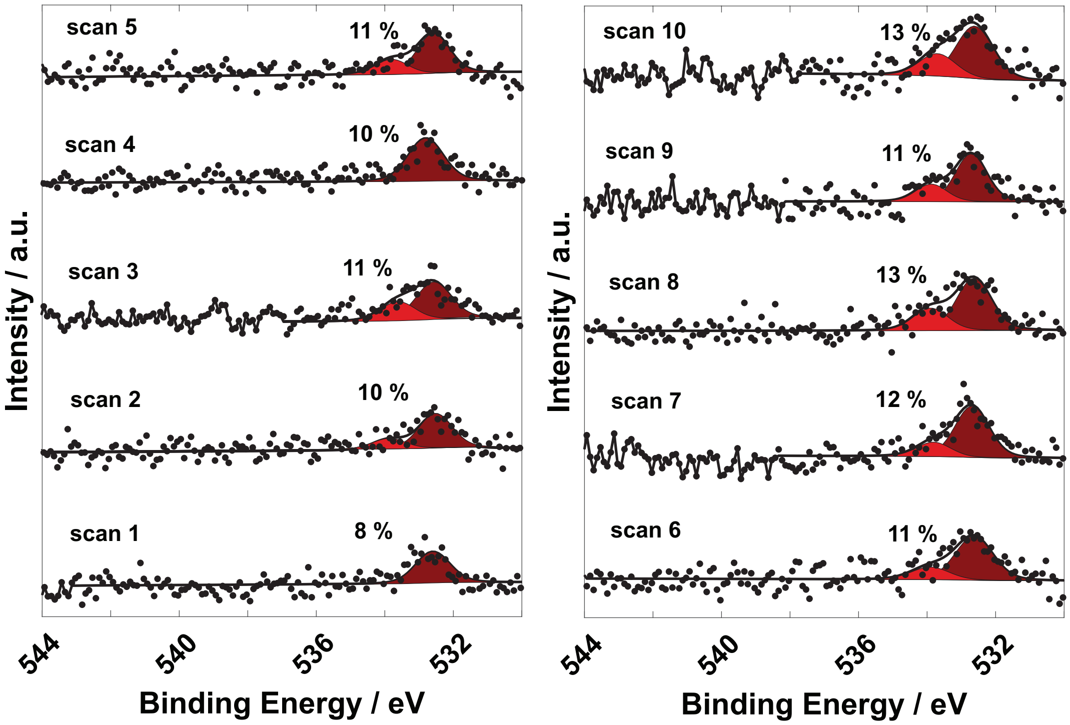

Figure S8 Individual (scan 1 to 10) O 1s high-resolution XPS core level spectra of a stoichiometric $Cs_{.05}FA_{.79}MA_{.16}$ film under 1×10^{-8} Torr that includes the O_2 gas binding energy region. The percentage of the O 1s elemental concentration is added for every scan as a function of exposure time.

moving under the influence of X-rays and the white light, but there is no other species present that can fill up the halide vacancy and chemistry is not observed.

The most acceptable corrosion model states that the anion vacancies near the central Pb atoms created due to the excitation process are passivated with $O_2^{-\cdot}$ species (with an oxidation state of -1/2) by breaking a Pb-I bond and forming two weaker O-Pb bonds, which explains the BE increase of the more weakly coordinated Pb state in scan 5.[33] Simultaneously, the BE of the O_2 species shifts to the 531-532 eV range, which is often related to lower coordinated O atoms with lower electron densities than the classical O_2^- ions found in the 530.5-531 eV region.[25] Further exposure to X-rays, white light and O_2 results in the buildup of more of these metastable Pb_{WC} atoms (scan 4 to 10 in Figure 2). We posit that the displaced iodide anions then migrate along the grain boundaries and are oxidized. In summary, Pb_{WC}-rich cathodic sites and I-rich anodic sites are formed and create an electrochemical gradient within the perovskite film to drive a cathodic reaction where photogenerated electrons residing close to the CBM generate superoxides, while the photogenerated hole in the VBM oxidizes mobile iodide anions.

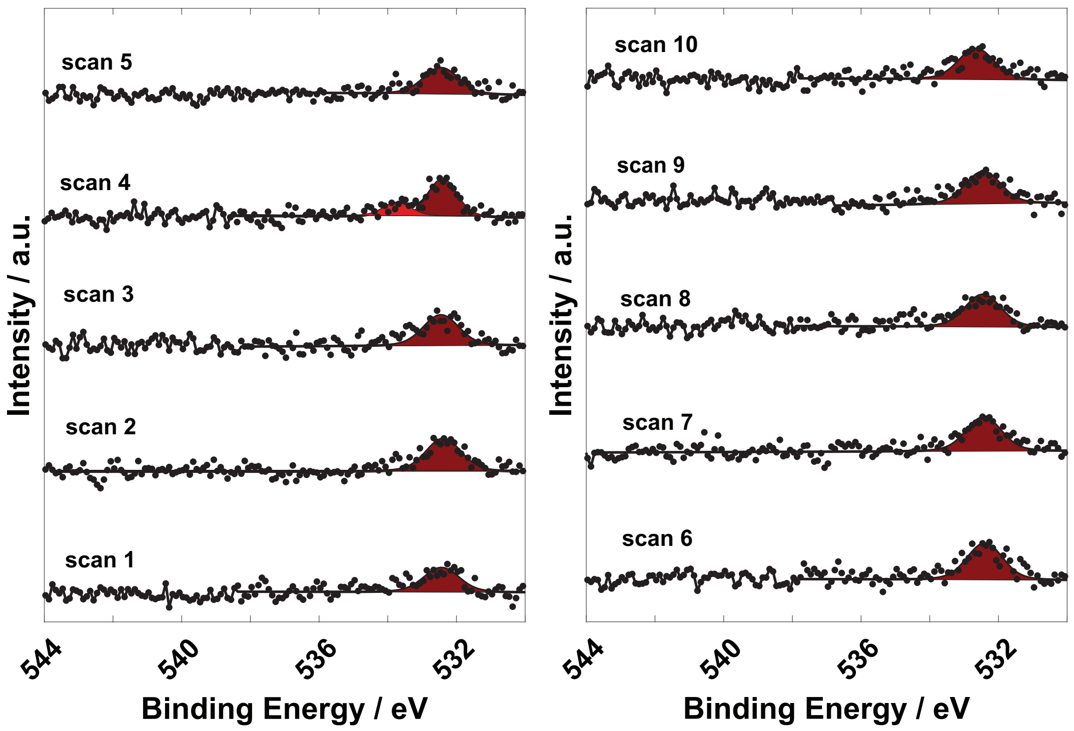

Figure S9 Individual (scan 1 to 10) O 1s high-resolution XPS core level spectra of a stoichiometric $Cs_{.05}FA_{.79}MA_{.16}$ film under 1×10^{-8} Torr after exposure to 2 mbar dry O_2 gas that includes the O_2 gas binding energy region. The percentage of the O 1s elemental concentration is added for every scan as a function of exposure time.

S7 Suppl. Note 4 - Why no decomposition of organic cations?

In previous literature, the decomposition of organic cations was proposed to be the most typical degradation pathway under X-ray and white light irradiation/superoxide.[6,7] To clarify this point, we have added the evolution of N 1s and C 1s high resolution core level spectra.

S7.1 N 1s high resolution core level spectra under gas exposure

The N 1s core level peak for MA is not visible in the scans in Figure S10 under O_2 exposure because the sensitivity is decreased when a gas is present in the measurement chamber and the concentration of MA in the triple cation is low. In general, the presence of O_2 gas makes it more difficult to pick up the signal of organic cations due to the loss in N 1s sensitivity.

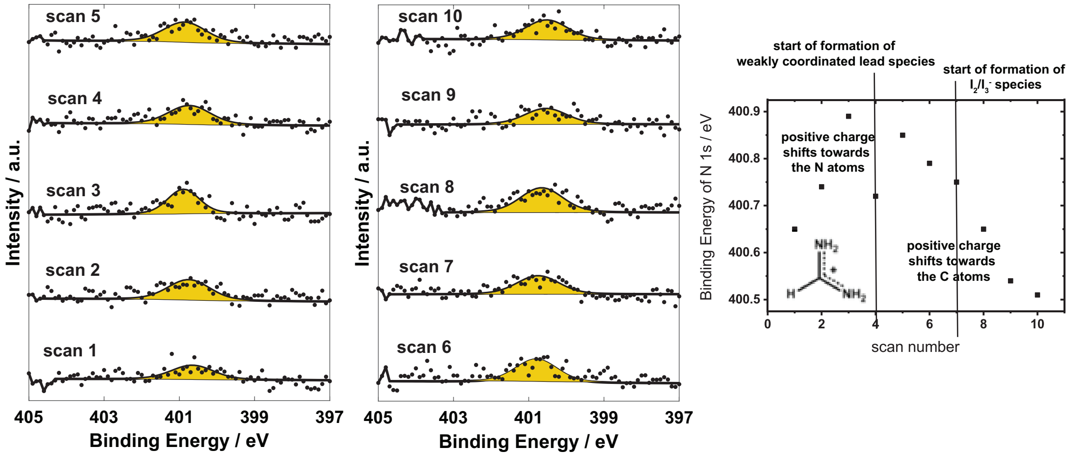

Figure S10 Individual (scan 1 to 10) N 1s high-resolution XPS core level spectra of a stoichiometric $Cs_{.05}FA_{.79}MA_{.16}$ film under 2 mbar dry O_2 gas in the measuring chamber and the dependence of the binding energy of N 1s core level peak as a function of the scan number.

The incorporation of superoxides ($O_2^{\cdot -}$), as suggested in the corrosion mechanism, would explain the small shifts of the positive charge on the N atoms of the FA cation because the interactions with the surrounding iodides are weakened. Iodides that have been dislodged from the crystal structure oxidize to I_3^-, but can also interact with the FA cation so that the positive charge shifts back to the C atoms.

S7.2 C 1s high resolution core level spectra under gas exposure

The C 1s high resolution core level spectra in Figure S11 shows that the C 1s peak for the FA cations is barely visible under O_2 exposure. The peak areas for both the adventitious carbon and the CO_x peak remain constant between 400 and 500, which means no other products are formed during the exposure of the perovskite to O_2 and X-rays. We see a slight drop in the C 1s peak areas than an

increase, which is more likely due to the loss of CO_2 and other adventitious carbon that was present before the sample was put in the XPS system.

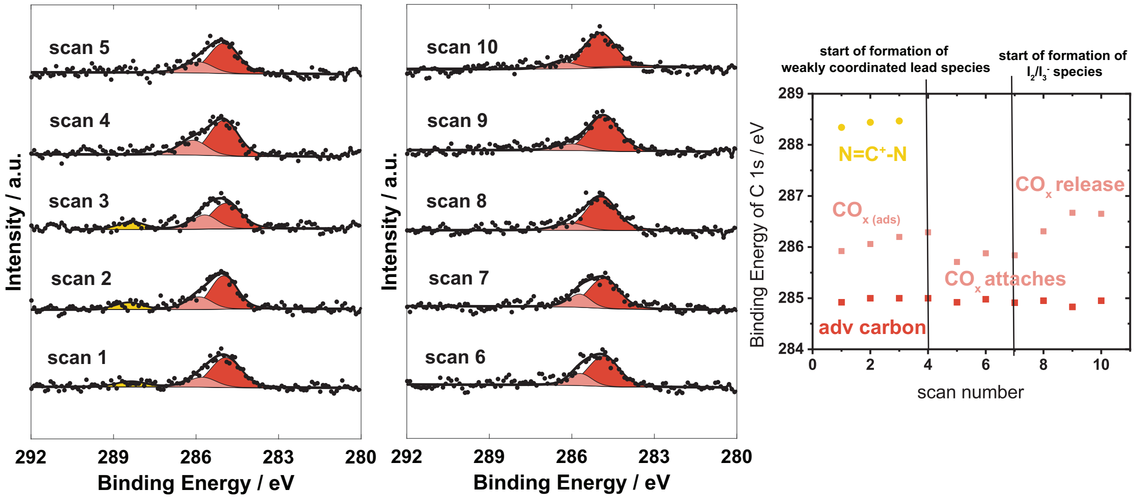

Figure S11 Individual (scan 1 to 10) C 1s high-resolution XPS core level spectra of a stoichiometric $Cs_{.05}FA_{.79}MA_{.16}$ film under 2 mbar dry O_2 gas in the measuring chamber and the dependence of the binding energy of C 1s core level peak as a function of the scan number.

S7.3 N 1s and C 1s high resolution core level spectra under UHV after gas exposure

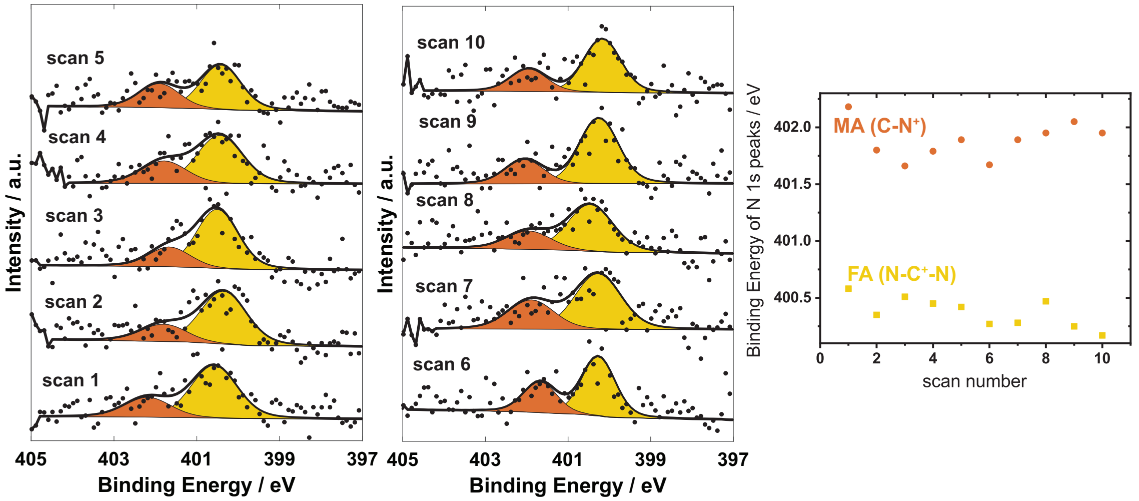

Figure S12 Individual (scan 1 to 10) N 1s high-resolution XPS core level spectra of a stoichiometric $Cs_{.05}FA_{.79}MA_{.16}$ film 1×10^{-8} Torr after exposure to 2 mbar dry O_2 gas and the dependence of the binding energy of N 1s core level peak as a function of the scan number.

The MA cation is still present under UHV in Figure S12 and the peaks have not disappeared after the reaction under O_2. The formation of an amine (like suggested by Aristidou et al.[7] based on theoretical data) starting from the ammonium in FA or MA would indicate a loss of the peak at

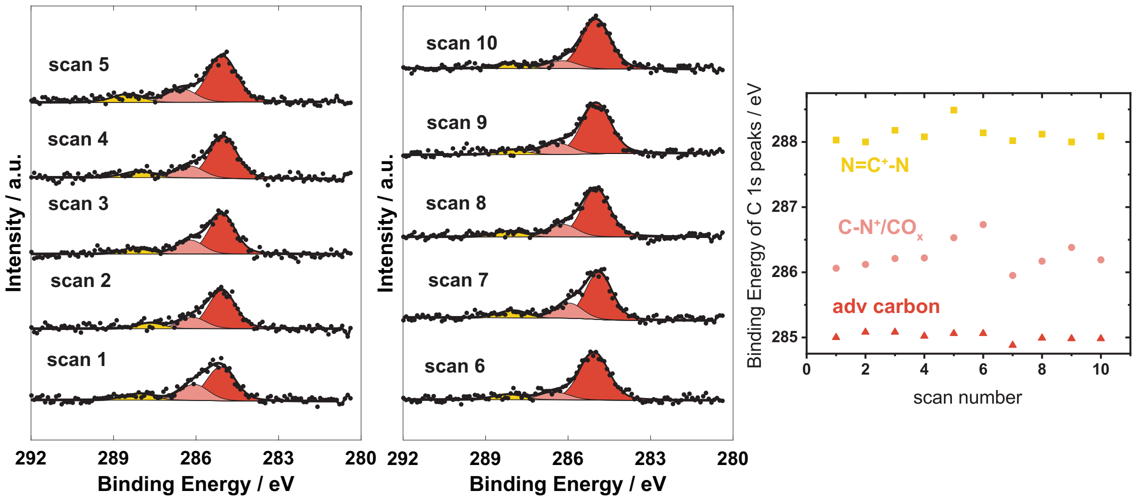

Figure S13 Individual (scan 1 to 10) C 1s high-resolution XPS core level spectra of a stoichiometric $Cs_{.05}FA_{.79}MA_{.16}$ film 1×10^{-8} Torr after exposure to 2 mbar dry O_2 gas and the dependence of the binding energy of C 1s core level peak as a function of the scan number.

$\approx$ 402 eV to form a peak around or below 400 eV. Both peaks remain stable across all subsequent scans demonstrating no direct evidence of degradation of the MA cations in these samples when exposed to X-rays and O_2. These scans suggest a change in counterion for the MA and FA cation, but certainly not a decomposition reaction. An increase in temperature might cause other reactions, but just the presence of O_2 and X-rays do not indicate organic cation decomposition. Furthermore, the C 1s high resolution spectra in Figure S13 under UHV after O_2 exposure indicate that FA cations are still present after reaction with O_2 and X-rays demonstrating no direct evidence of degradation of the FA cations. Our main hypothesis for the stability of the organic cations (i.e., methylammonium) in the perovskite film under study is the absence of water in this experiment. Presumably, following the Brønsted-Lowry definition, water acts as a proton acceptor (or base) during the start of the decomposition reaction, while the organic cation acts as a proton acceptor (or weak acid) as described below in Equation (S3). This would suggest that water is necessary component during organic cation degradation following this acid-base type reaction and certainly not the same principles as the redox reaction described in our model.

$$CH_3NH_3^+ + H_2O \rightleftharpoons CH_3NH_2 + H_3O^+ \tag{S3}$$

By removing water from the aging environment in this experiment, we have been able to shut off this degradation pathway for the organic cations. Of course, this theory needs to be further tested in future studies.

S8 Suppl. Note 5 - Photoelectrochemical reaction mechanism

Table S5 Photochemical reaction mechanism and rate equations for the reaction between a perovskite film and dry O_2.

Reaction	Reaction Rate Equation
$\text{perovskite} + O_{2,(\text{g - 2 mbar})} \rightleftharpoons \text{perovskite}(O_{2,\text{ads}})$ (S4)	weak (equilibrium) adsorption
$\text{perovskite}(O_{2,\text{ads}}) + \text{h}\nu \overset{k_1}{\rightleftharpoons} \text{perovskite}(O_{2,\text{ads}})^*$ (S5)	$v_c^{(1)} = -\frac{\partial[\text{perovskite}(O_{2,\text{ads}})]}{\partial t} = -\frac{\partial[h\nu]}{\partial t} = \frac{\partial[\text{perovskite}(O_{2,\text{ads}})^*]}{\partial t} = k_1[\text{perovskite}(O_{2,\text{ads}})][h\nu]$
$\text{perovskite}(O_{2,\text{ads}})^* \overset{k_2}{\rightleftharpoons} \text{perovskite}(O_2^{-\cdot}/\text{Pb}_{\text{WC}}) + \text{I}^-_{\text{gb,mobile}} + \text{h}^+$ (S6)	$v_c^{(2)} = -\frac{\partial[\text{perovskite}(O_{2,\text{ads}})^*]}{\partial t} = \frac{\partial[\text{perovskite}(O_2^{-\cdot})]}{\partial t} = \frac{\partial[\text{I}^-]}{\partial t} = k_2[\text{perovskite}(O_{2,\text{ads}})^*]$
$\text{perovskite}(O_{2,\text{ads}})^* \overset{k_3}{\rightleftharpoons} \text{perovskite} + h\nu'$ (S7)	$v_c^{(3)} = -\frac{\partial[\text{perovskite}(O_{2,\text{ads}})^*]}{\partial t} = \frac{\partial[\text{perovskite}]}{\partial t} = k_3[\text{perovskite}(O_{2,\text{ads}})^*]$
$\mathbf{perovskite(O_{2,ads}) + h\nu \rightleftharpoons perovskite(O_2^{-\cdot}) + I^-_{gb,mobile} + h^+}$ (S8)	

The reaction sequence (see also section 2.1 in main paper) is initiated by the weak adsorption of oxygen to the perovskite in Equation (S4). Given the constant partial pressure of O_2, and the anticipated fast diffusion through the perovskite thin film, we assume a steady-state concentration of perovskite($O_{2,\text{ads}}$) throughout the near surface illuminated region as proposed by Prezhdo and coworkers.[34] Photon absorption following the Stark-Einstein law as described in Equation (S5) pre-

dicts that, for each photon of light absorbed by a chemical system, only one molecule is activated for the subsequent reaction, which means the reaction is dependent on the accumulated photon dose.[35] For the assumption that adsorbed oxygen species are present, we refer to the changes in the O/Pb ratio. The excited perovskite site (i.e., perovskite$(O_{2,ads})^*$ – a bound 'electron–hole pair') then acts as a source of electrons and gives an excited electron present in the CBM to the adsorbed O_2 to form a one-electron adduct of molecular oxygen or a superoxide $(O_2^{-\cdot})$ in Equation (S6), which is still adsorbed as perovskite$(O_2^{-\cdot})$. We anticipate that superoxide and/or other negative charged forms of oxygen (including 1/2 O_2^{2-}) weaken the Pb-I interaction, dislodging an iodide anion from the PbI_6^{4-} octahedron to move to the grain boundaries (i.e., $I^-_{gb,mobile}$) to leave a more weakly coordinated Pb atom. The photogenerated hole (h^+) is free to react in close proximity, initiating the corrosion process. In a competitive process, the excited perovskite site can also be quenched by spontaneous emission of a photon hν' (different wavelength; different propagation direction) or by nonradiative recombination as shown in Equation (S7). From a kinetics perspective, the quantity measured in the XPS experiment is the rate of product formation $\frac{\partial[\text{perovskite}(O_2^{-\cdot})]}{\partial t}$, which is $v_c^{(3)} = k_3[\text{perovskite}(O_{2,ads})^*]$ according to Equation (S8). Therefore, the rate expression involving $[\text{perovskite}(O_{2,ads})^*]$, is pertinent, $\frac{\partial[\text{perovskite}(O_{2,ads})^*]}{\partial t} = k_2[\text{perovskite}(O_{2,ads})][h\nu]$, which depends on the steady-state concentration $[\text{perovskite}(O_{2,ads})]$. Based on a diffusion coefficient of O_2 of ca. 10^{-8} cm^2 s^{-1} within $MAPbI_3$ perovskites,[7] we note that O_2 saturation for these perovskite films is predicted to be instantaneous, which means the 'reaction depth' is determined by the penetration of electromagnetic radiation (1486.6 eV X-rays) governed by the absorption coefficient, estimated to be 10^3 – 10^4 cm^{-1}.[7] Here, we assume a quasi-steady-state condition for $[\text{perovskite}(O_{2,ads})^*]_{SS}$, where the population of perovskite$(O_{2,ads})^*$ is negligible and deactivation by the quenching reactions (i.e., Equation (S6)) is much faster than its excitation process in Equation (S5). Thus, for $\frac{\partial[\text{perovskite}(O_{2,ads})^*]_{SS}}{\partial t} = 0$, we obtain $[\text{perovskite}(O_{2,ads})^*]_{SS} = \frac{k_1[\text{perovskite}(O_{2,ads})][h\nu]}{k_2+k_3}$ which leads to

$$v_c = \frac{k_1k_2[\text{perovskite}(O_{2,ads})][h\nu]}{k_2+k_3} = \frac{k_1k_2}{k_2+k_3}[\text{perovskite}(O_{2,ads})][h\nu] \quad \text{(S9)}$$

Following Equation (S9), this overall process is second order, however, $[\text{perovskite}(O_{2,ads})]$ is only slightly diminishing because of the large number of excitable sites within the bulk perovskite material, the net behavior is that of a pseudo first order reaction. Additionally, the photon dose is

constant during the X-ray exposure, so that the apparent order becomes zero as demonstrated in Figure 2C. This means the effective rate is constant during the reaction because the photon dose is kept constant. Under these circumstances, reaction rate is predicted to scale with light fluence. We emphasize that this proposed photoelectrochemical reaction is second order but that the apparent order can vary dependent on the initial conditions, i.e. decreasing the oxygen partial pressure or X-ray beam fluence will, although the true order of the reaction remains second order.

S9 Suppl. Note 6 - Origin of strongly coordinated Pb atoms

X-ray diffraction and X-ray photoelectron studies of various degraded iodide-based perovskite samples have shown characteristic peaks for the formation of lead iodide, PbI_2, for light-, heat- and moisture-induced degradation.[36–38] Therefore, the first obvious choice is to identify the BE shifts for PbI_2 using two-dimensional plots combining the BEs of the core level transitions and the X-ray-induced Auger lines for the Pb atom.[19] The UHV high-resolution Pb 4f core level spectra (A) and the Pb $N_7O_{4,5}O_{4,5}$ and $N_6O_{4,5}O_{4,5}$ Auger lines (B) for a PbI_2 film on an ITO substrate, kept under inert atmosphere and under air, deposited using the same procedure as described in section 1.1 is demonstrated in Figure S14. The charge-shift corrected BE of the Pb $4f_{7/2}$ peak for the PbI_2 film is measured at 138.2 eV in Figure S14A and reflects the same BE as for the corner-sharing $[PbI_6]^{4-}$ octahedra in the perovskite film. We also observe weakly coordinated Pb atoms in the near-surface region of the PbI_2 film (≈2%), which is indicative of a more electroneutral (i.e., more electron rich) central Pb atom. Solvent molecules are prone to compete with iodide to coordinate with Pb^{2+} to form different types of iodoplumbate complexes in solution.[29] Solutions containing PbI_2/DMF, not containing an excess of iodide, exhibit significant DMF coordination,[30] where DMF coordinates to PbI_2 through the formation of PbO bonds, creating $PbI_2 \cdot$ DMF complexes, which explains the increased electron-richness around the central Pb atom.[31] When the film is exposed to air, these weakly coordinated sites quickly oxidize with the formation of a more coordinated central Pb atom as demonstrated in Figure S14A. In Figure S14B, the KE of the Pb $N_6O_{4,5}O_{4,5}$ Auger line shows a pronounced shift from 93.9 eV to 93.49 eV when the PbI_2 film is exposed to air, which confirms that the coordination state of the central Pb atoms in the near-surface region has changed. However, PbI_2 cannot be the origin for the high BE shoulder seen in Figure 4A.

Because we analyze mixed-halide perovskite films, we need to consider the presence of bromide and the formation of iodide-rich and bromide-rich domains as previously proposed during degradation processes.[21,39,40] The UHV high-resolution Pb 4f core level spectra (A) and the Pb $N_7O_{4,5}O_{4,5}$ and $N_6O_{4,5}O_{4,5}$ Auger lines (B) for a $PbBr_2$ film on an ITO substrate, that represents bromide-rich domains, deposited using the same procedure as described in section 1.1 is demonstrated in Figure S14. The BE of the Pb $4f_{7/2}$ peak for the $PbBr_2$ film is measured at 138.85 eV in Figure S14A. This charge-shift corrected BE change, compared to the PbI_2 film, is explained by the dipole moment differences (and the overall polarizability differences) along the Pb-I bond in PbI_2 (μe = 4.3 D) and the Pb-Br bond (μe = 5.0 D) in $PbBr_2$.[41] The larger dipole moment along the Pb-Br bond creates

more electron-poor (or strongly coordinated) Pb atoms within the perovskite film. The $N_6O_{4,5}O_{4,5}$ Auger lines in Figure S14B show a more pronounced shift to a lower KE (or higher BE), namely from 93.9 eV to 92.8 eV. We therefore propose that the origin for the high BE shoulder seen in Pb 4f spectra Figure 4A is related to the formation of bromide-enriched $[PbX_6]^{4-}$ octahedra in the near-surface 'corrosion cell' region.

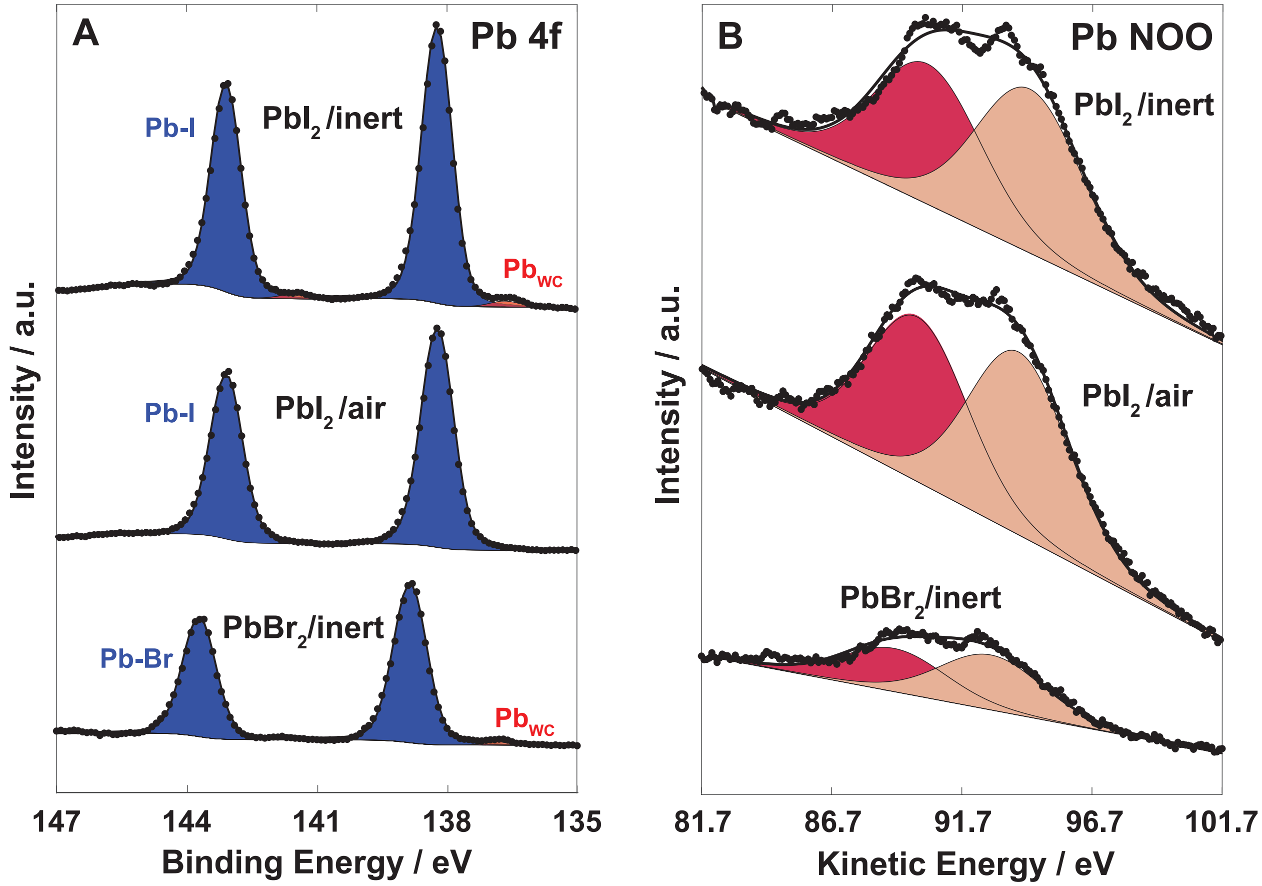

Figure S14 (A) High-resolution Pb 4f core level spectra and (B) Pb NOO Auger lines for a PbI_2 film exposed to only an inert atmosphere, a PbI_2 film exposed to air and a $PbBr_2$ film exposed to only an inert atmosphere.

S10 Gain and loss of strongly coordinated Pb atoms

Table S6 Calculated (or expected composition from precursor solution) and measured relative atomic ratios using the Scofield RSFs from the high-resolution Pb 4f, I 3d, Cs 3d, Br 3d, N 1s and C 1s core level spectra for the stoichiometric $Cs_{.05}FA_{.79}MA_{.16}$ films.

Ratio	stoichiometric $Cs_{.05}FA_{.79}MA_{.16}$ (calculated)	stoichiometric $Cs_{.05}FA_{.79}MA_{.16}$ (not degraded; step I)	stoichiometric $Cs_{.05}FA_{.79}MA_{.16}$ (degraded; step III)
I/Pb	2.61	3.75[1]	3.34
Cs/Pb	0.05	0.06	0.02
Br/Pb	0.39	0.35	0.42
N/Pb	1.74	2.15	1.89
FA(N)/Pb	1.58	1.89	1.68
MA(N)/Pb	0.16	0.26	0.66
C/Pb	0.95	2.35	4.69
MA(C)/Pb	0.16	1.17	2.87
FA(C)/Pb	0.79	1.17	1.13
O/Pb	-	1.54	3.46

[1]increased I/Pb ratio due the iodide surface enrichment

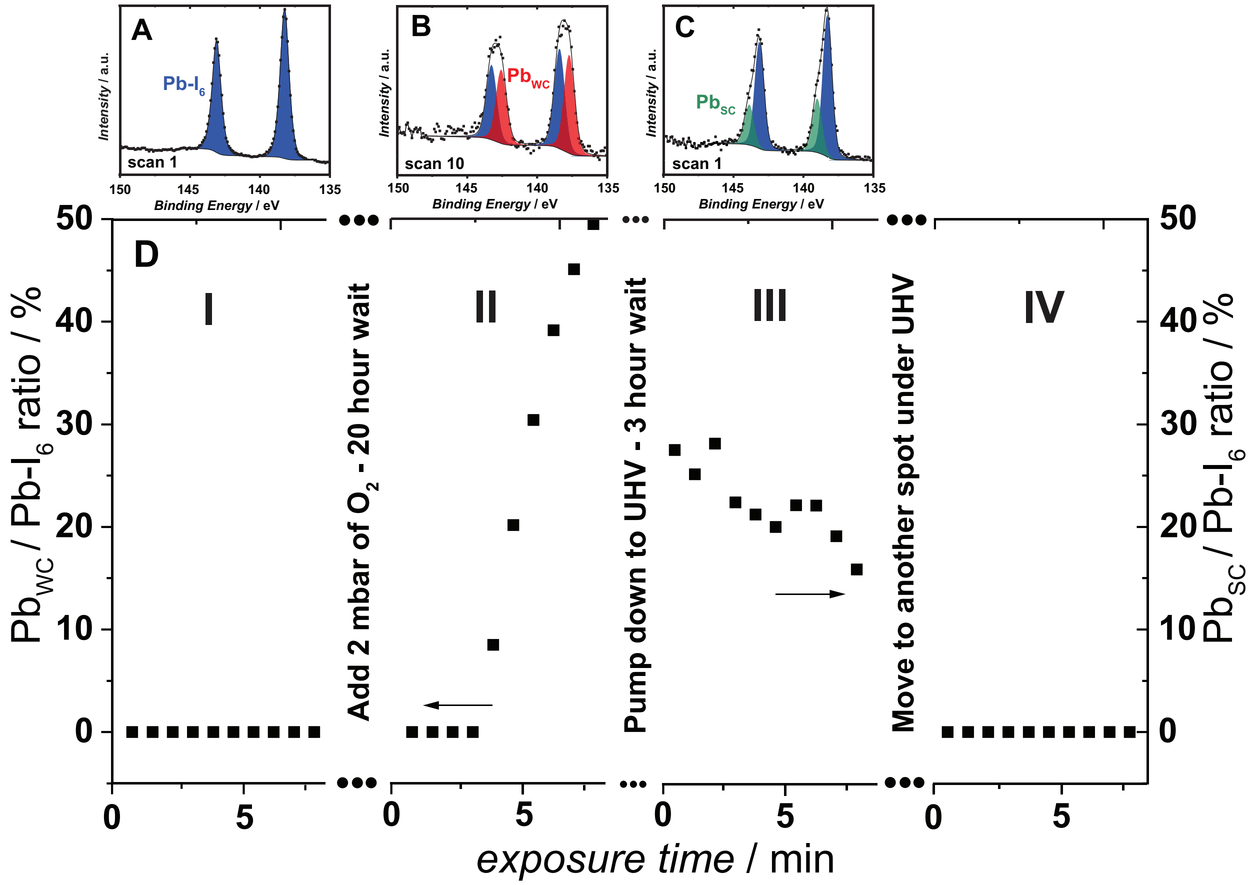

Figure S15 (A-C) High-resolution Pb 4f XPS data, and time dependencies for formation of Pb_{WC} and Pb_{SC} for the stoichiometric perovskite $Cs_{.05}FA_{.79}MA_{.16}$ (A) under vacuum (only the $Pb\text{-}I_6$-like form observed); (B) same analysis spot during exposure to O_2/X-rays/white light - Pb_{WC}/Pb_{total} rapidly increases, (C) same analysis spot after return to UHV conditions Pb_{WC} disappears and is replaced by Pb_{SC}; (D) Time dependencies of Pb_{WC}/Pb_{total} and Pb_{SC}/Pb_{total} for conditions in (A-C). The last panel shows Pb_{WC}/Pb_{total} for a second analysis spot (under UHV conditions) that had been exposed to dry O_2 gas during (A-C), but not under X-ray illumination Pb 4f line shapes were unchanged from the as-deposited perovskite, with only one Pb form observed.

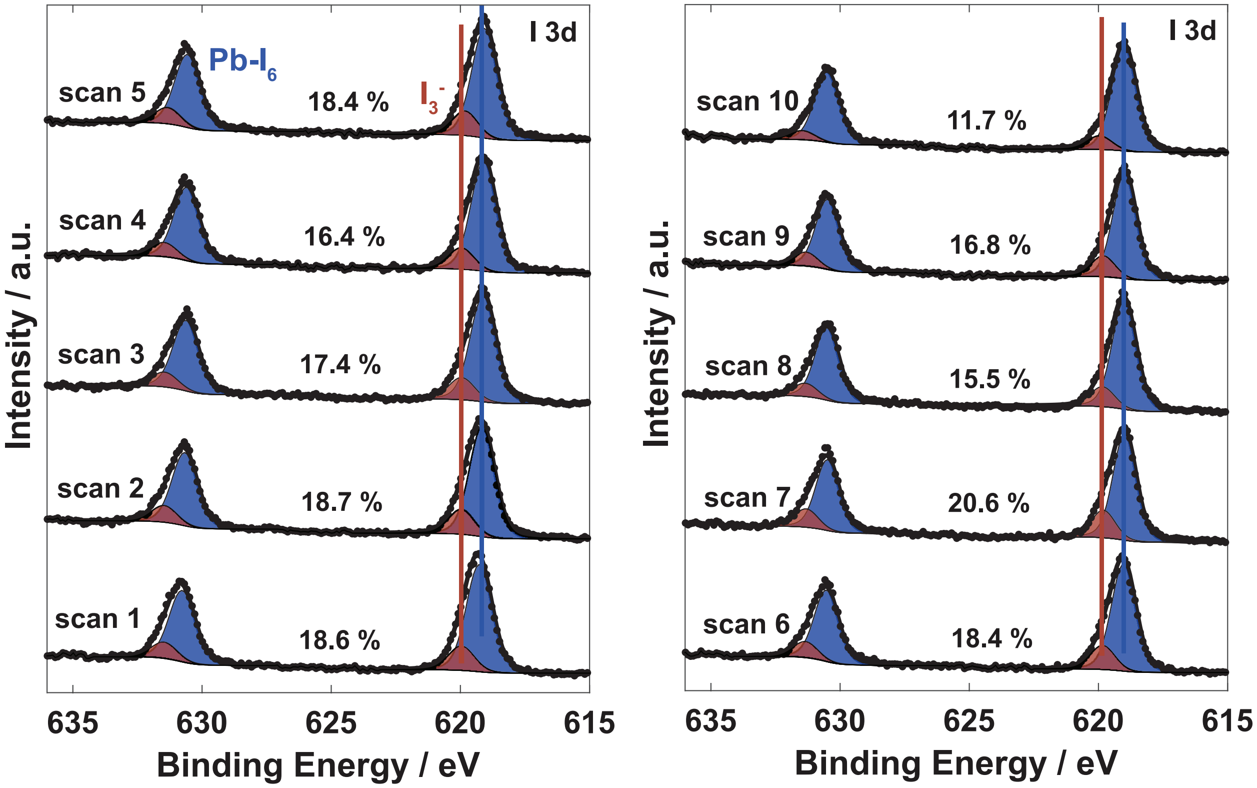

Figure S16 The corresponding high resolution I 3d core level spectra showing the gradual loss of higher BE peaks ascribed to I_3^- as the coordination environment of the stoichiometric perovskite is restored.

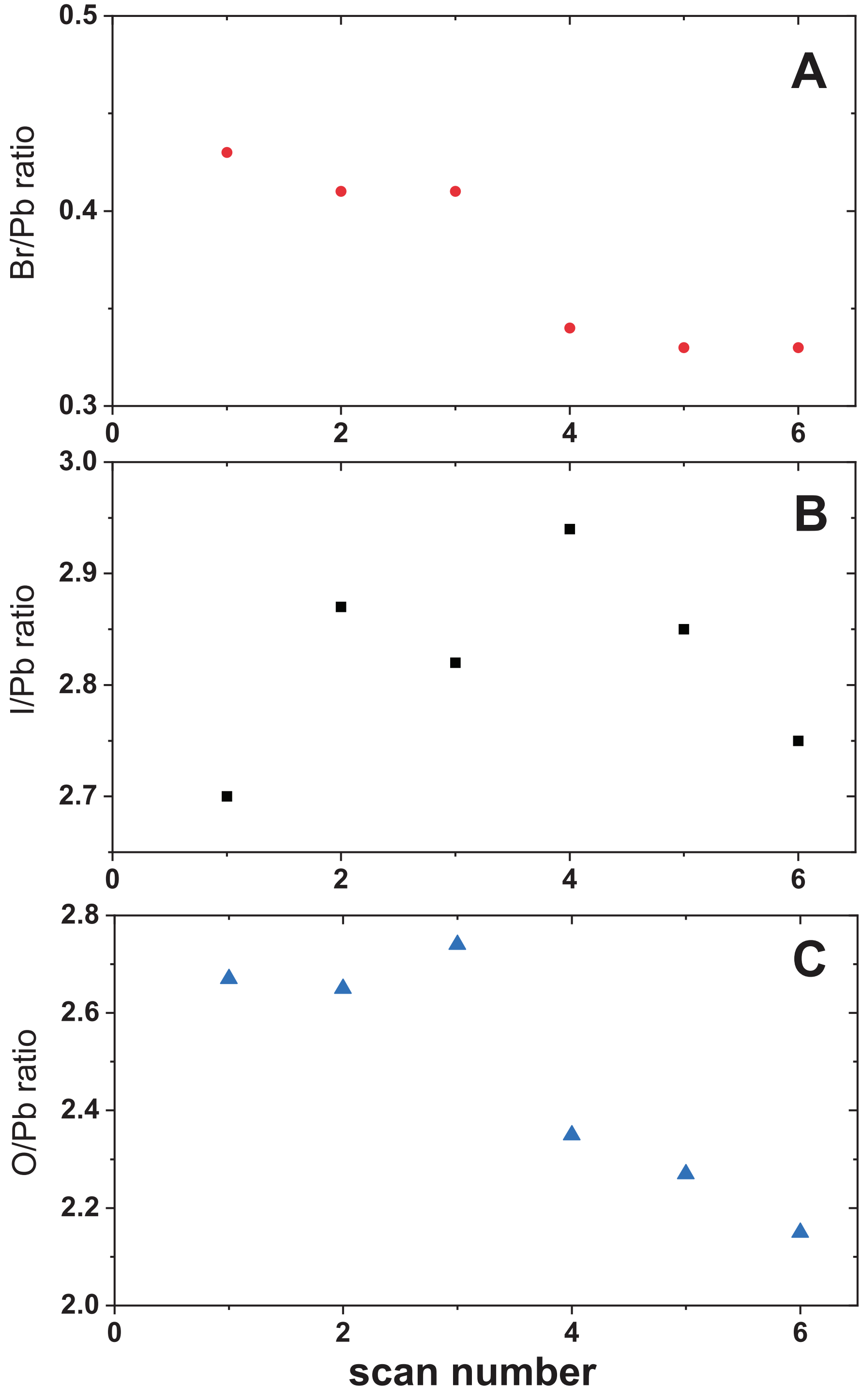

Figure S17 Time-dependent (A) Br/Pb, (B) I/Pb and (C) O/Pb relative atomic ratios as a function of scan number calculated using the RSFs in Table S3 under UHV after O_2 and light exposure (Experiment III).

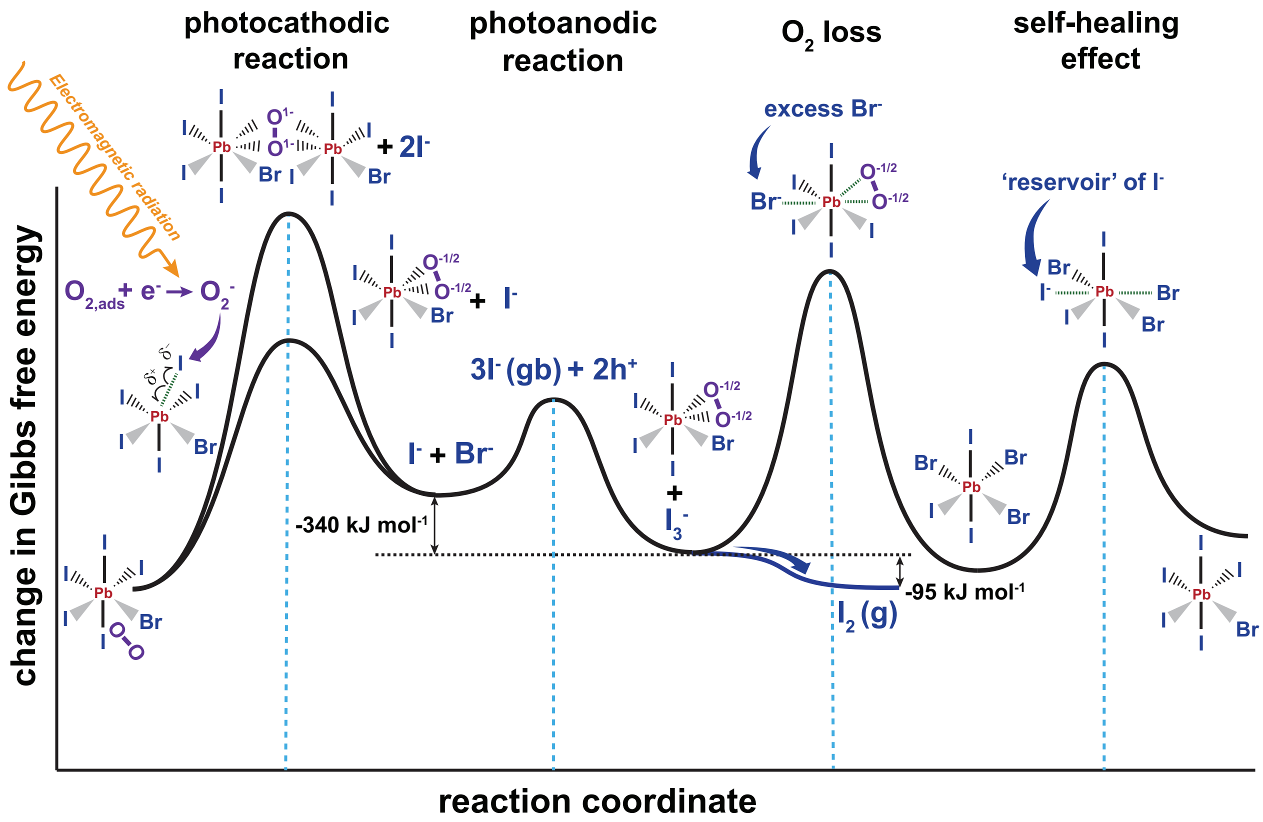

Figure S18 Schematic representation of the ligand exchange process in the corner-sharing $[PbX_6]^{4-}$ octahedra that describes the active corrosion mechanism for mixed halide perovskite films.

S11 Suppl. Note 7 - Degradation of FAI-rich CsFAMA perovskites

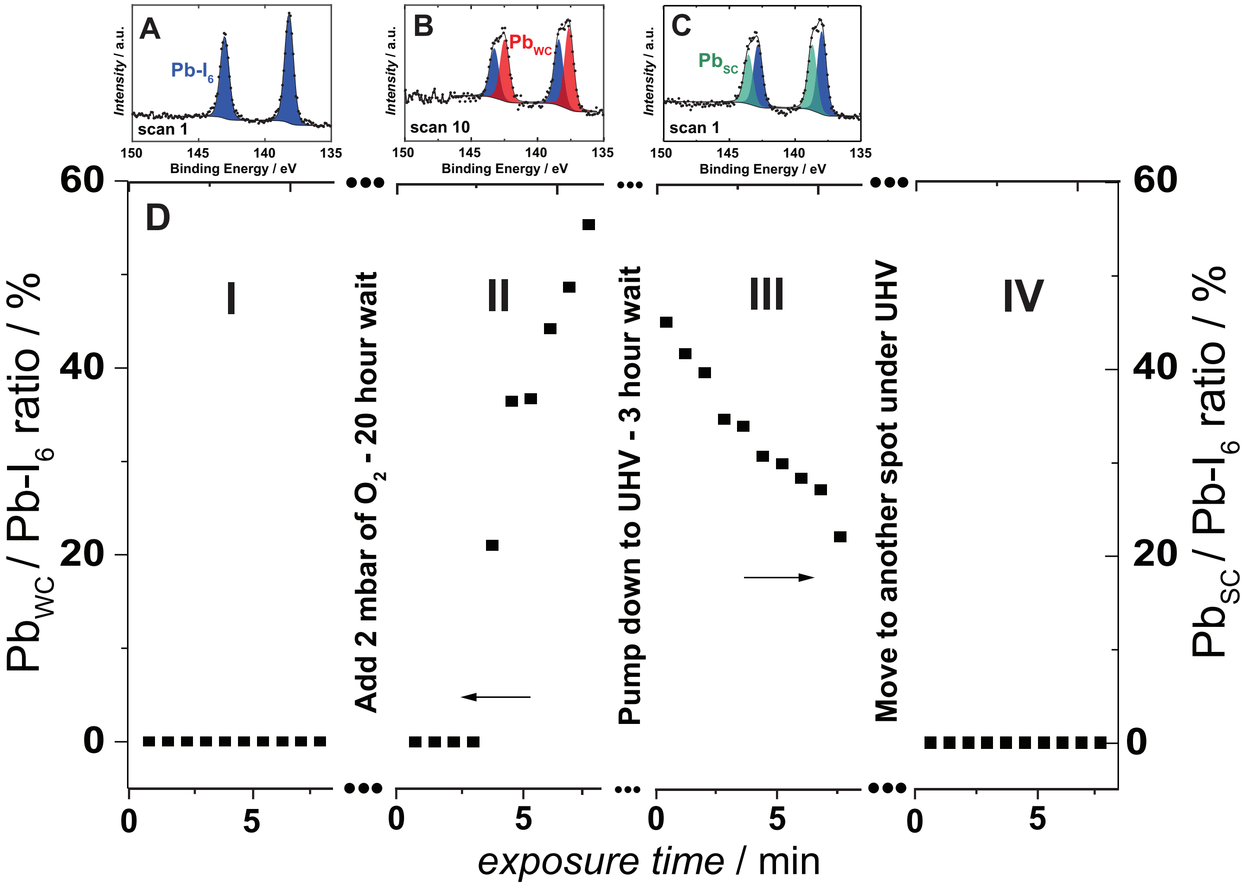

Figure S19 (A-C) High-resolution Pb 4f XPS data, and time dependencies for formation of Pb_{WC} and Pb_{SC} for the FAI-rich perovskite $Cs_{.05}FA_{.83}MA_{.16}$ (A) under vacuum (only the $Pb\text{-}I_6$-like form observed); (B) same analysis spot during exposure to O_2/X-rays/white light - Pb_{WC}/Pb_{total} rapidly increases, (C) same analysis spot after return to UHV conditions Pb_{WC} disappears and is replaced by Pb_{SC}; (D) Time dependencies of Pb_{WC}/Pb_{total} and Pb_{SC}/Pb_{total} for conditions in (A-C). The last panel shows Pb_{WC}/Pb_{total} for a second analysis spot (under UHV conditions) that had been exposed to dry O_2 gas during (A-C), but not under X-ray illumination Pb 4f line shapes were unchanged from the as-deposited perovskite, with only one Pb form observed.

Similarly, to stoichiometric compositions, FAI-rich compositions undergo photoelectrochemical reactions Equations (S4) to (S8) to form weakly coordinated Pb atoms next to Pb atoms coordinated in corner-sharing $[PbI_6]^{4-}$ octahedra as demonstrated in the high-resolution Pb 4f core level scans in Figure S19A (blue peak) and the lower BE shoulder at 137.8 eV in Figure S19B (red peak). After O_2 is removed, the higher BE shoulder at 138.8 eV in Figure S19C correlates with the formation of strongly coordinated Pb atoms (green peak). Figure S19D demonstrates an increased growth rate of the relative ratio of Pb_{WC} atoms following zero-order kinetics as demonstrated in Figure S20 following (A) the time-dependent decay of the $Pb\text{-}I_6$ coordination peak and (B) the growth of the

Pb_{WC} atom coordination peak. The effective rate constant for the photoelectrochemical degradation of the FAI-rich triple cation perovskite in the near-surface region is 2.86 × 10^{-4} at%($Pb\text{-}I_6$) s^{-1} and corresponds to a half-life time, $t_{1/2}$, of 4.9 minutes, which is faster than the 5.5 minutes measured for the stoichiometric perovskite film as summarized in Table 2.

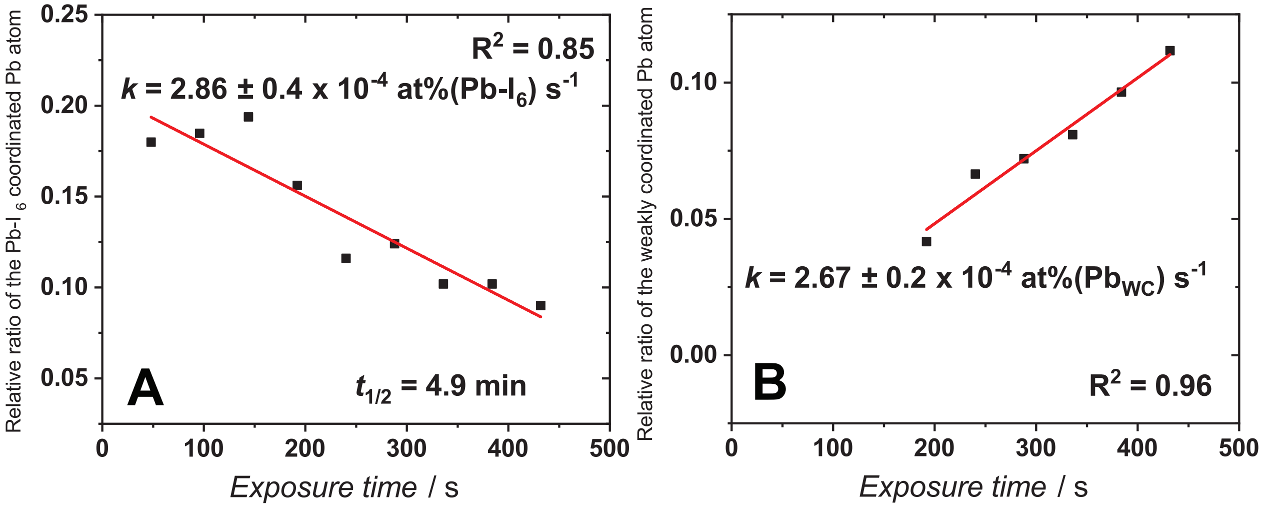

Figure S20 (A) Atomic percentage of the $Pb\text{-}I_6$ coordinated Pb to the total atomic percentage that includes all lead, iodide, and bromide vs. the exposure time and (B) Atomic percentage of Pb_{WC} to the total atomic percentage that includes all lead, iodide, and bromide vs. the exposure time. Both use the Pb 4f, I 3d and Br 3d high-resolution XPS core level spectra for the FAI-rich perovskite film measured under 2 mbar O_2 under illumination.

Because the photon dose is the same in both cases, the increase in half-life time needs to be related to the rate of superoxide formation. Electronically, this means that the FAI-enriched triple cation perovskite must have donor defect energy levels that are better aligned with the reduction potential for the $O_2/O_2^{\cdot-}$ reaction, which is ca. -0.55 V vs Fc/Fc^+ (or -4.25 eV vs local vacuum level) in standard conditions.[42] Looking at Figure 5, based on the donor defects quantified in our previous spectroelectrochemical studies,[12] this is explained by the presence of deeper donor defects within the FAI-rich perovskite compared to the stoichiometric one.

Next to the formation of Pb_{WC} atoms, we know that the concentration of iodide ions in the grain boundaries in the illuminated near-surface region increases, but instead of a photoanodic process with the formation of oxidized iodide-based species, we notice the growth of lower BE shoulder in the time-dependent high resolution I 3d core level spectra as demonstrated in Figure S21. Here, the purple peak with a BE of ca. 619.1 eV represents the iodide atom in corner-sharing $[PbI_6]^{4-}$ octahedra (called $Pb\text{-}I_6$), while the new peak with a maximum BE of ca. 618.5 eV appears after ± 4.8 minutes (i.e., the time to take 6 scans) and is best correlated with the presence of alkali metal

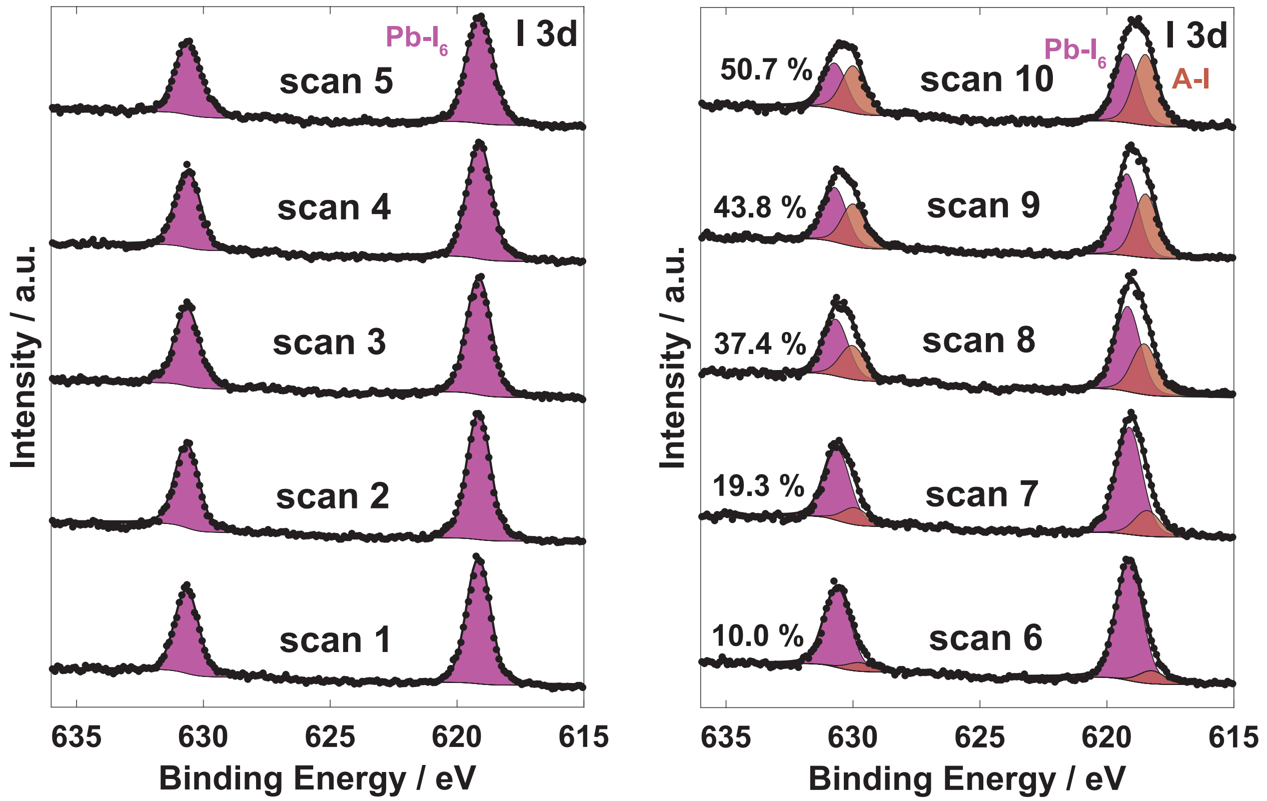

Figure S21 Individual (scan 1 to 10) I 3d high-resolution XPS core level spectra of a FAI-enriched triple cation perovskite film under dry O_2 gas exposure and white light in the measuring chamber of the XPS instrument. The percentages indicate the relative amount of iodide in the form of A-I compared to the total amount of iodide.

iodides.[43] More correctly, this new peak describes the strong ionic bond between excess cations and iodide anions, which retards the direct generation of oxidized iodide species.

Interestingly, only when the dry O_2 gas is completely removed in Figure S22, the excess of iodide at the illuminated perovskite surface is oxidized using the excess of presumably long-lived photogenerated holes to form triiodide species. Because the near-surface region is first heavily enriched with iodide anions when O_2 is present, very high triiodide concentrations are measured at the perovskite surface seen in scan 1 in Figure S22. When the sample is then systematically illuminated with X-rays under UHV, iodide anions start to migrate again so that the excess triiodide is reduced, which explains the I_3^- peak decrease at a BE of 619.8 eV. Similarly, as for the stoichiometric perovskite film, both the surrounding perovskite material and reduced I_3^- could act as an iodide feed to restore the bromide enriched $[PbX_6]^{4-}$ octahedra, which causes the increased restoration rate through the loss of strongly coordinated, Pb_{SC}, atoms described in Figure 4E.

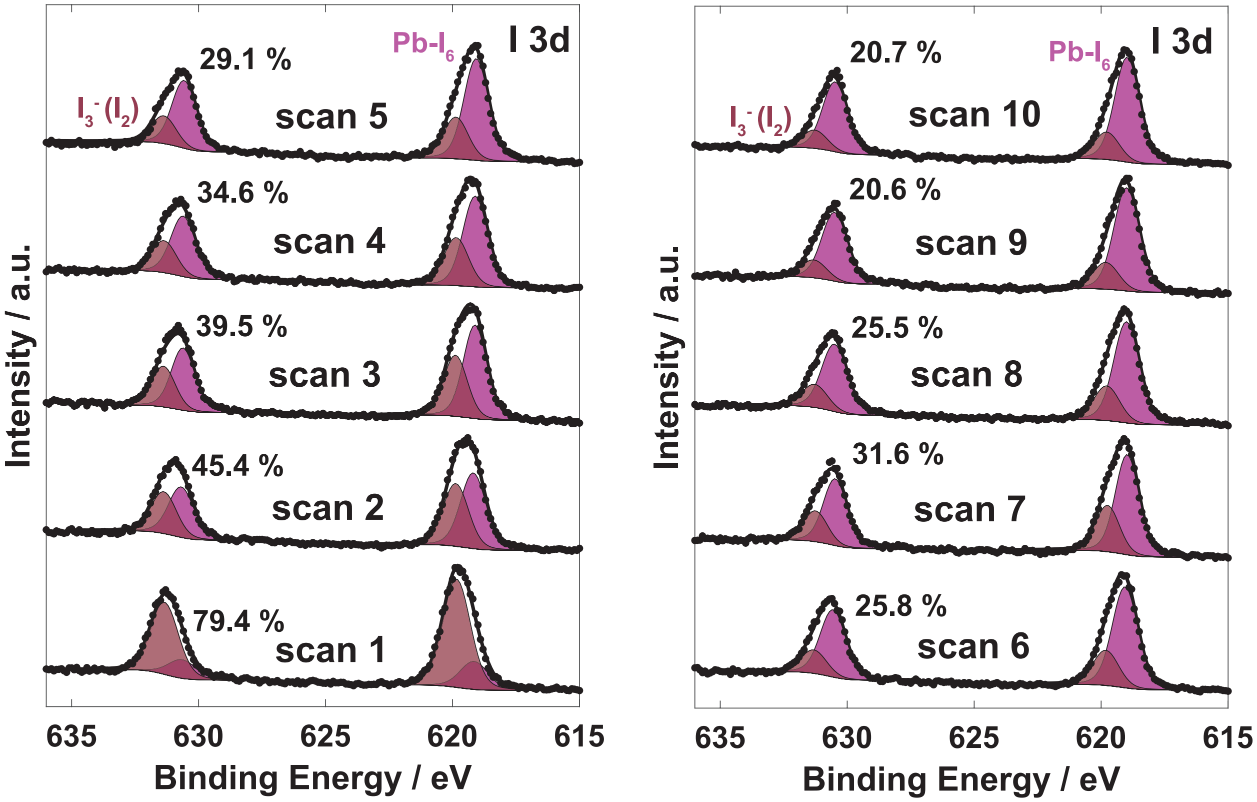

Figure S22 Individual (scan 1 to 10) I 3d high-resolution XPS core level spectra of a FAI-enriched triple cation perovskite film under UHV and white light after dry O_2 gas exposure. The percentages indicate the relative amount of oxidized iodide in the form of I_3^- (or chemisorbed I_2) compared to the total amount of iodide.

S12 Suppl. Note 8 - Degradation of PbI_2-rich/FAI-deficient CsFAMA perovskites

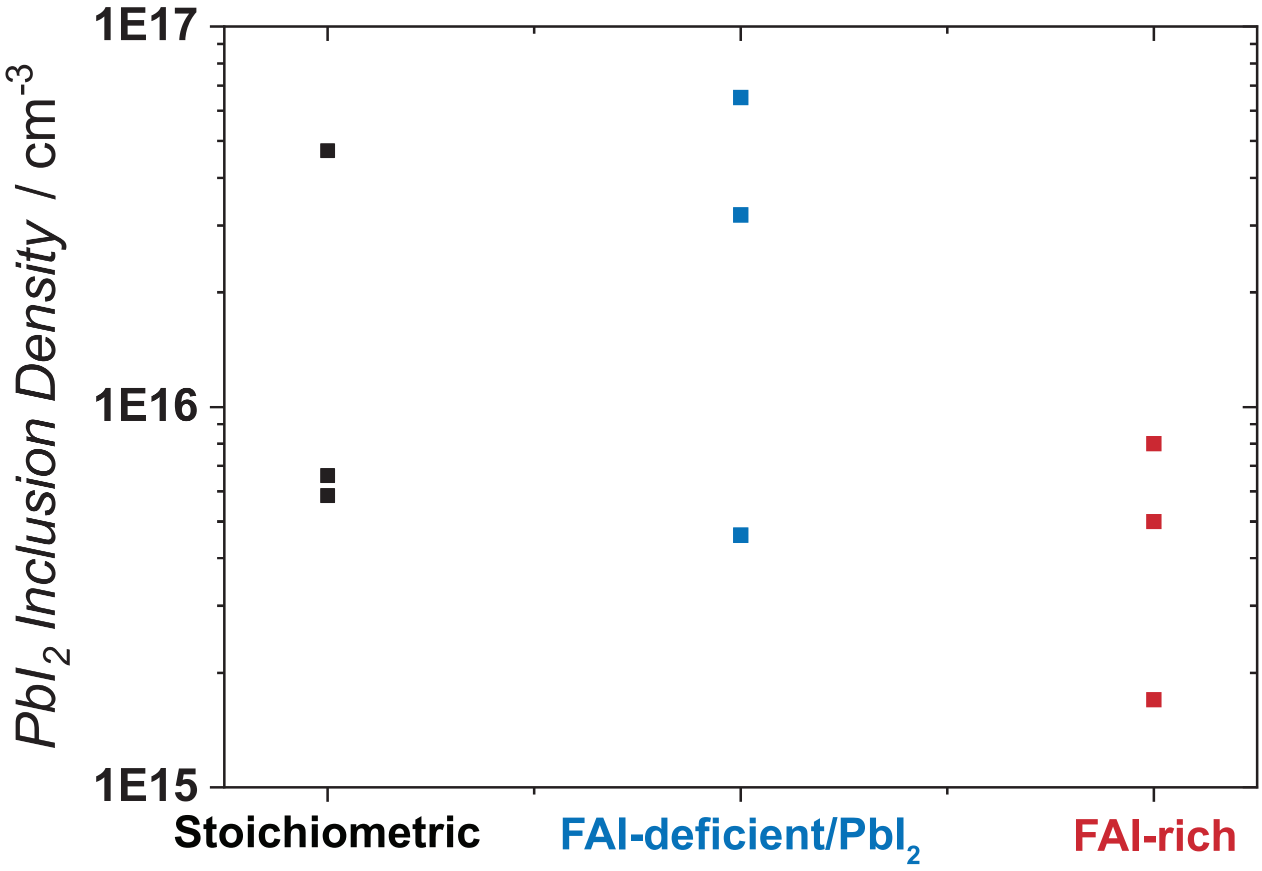

Figure S23 PbI_2 inclusion density and its heterogeneity from the STEM images of stoichiometric $Cs_{.05}FA_{.79}MA_{.16}$ and, FAI-deficient/PbI_2-rich $Cs_{.05}FA_{.75}MA_{.16}$ and FAI-rich $Cs_{.05}FA_{.83}MA_{.16}$ films.

For FAI-deficient/PbI_2-rich triple cation perovskites, XRD data in Figure S1 and the analysis of multiple preliminary STEM-HAADF images show a somewhat higher density of PbI_2 inclusions compared to the stoichiometric film, as summarized in Figure S23. In contrast a lower density of PbI_2 inclusions was observed for FAI-rich perovskites. For FAI-deficient/PbI_2-rich triple cation perovskites, the iodide deficiency within the initial composition (i.e., less FAI precursor is added) ensures an excess of bromides is present within the grain boundaries to compete with the superoxides from the start of the experiment. The iodide deficiency is also explained through the lack of acceptor defect states within the FAI-deficient/PbI_2-rich film as shown in in our previous spectroelectrochemical studies.[12] We propose that this excess of bromides ensures that Pb atoms coordinated in corner-sharing $[PbI_6]^{4-}$ octahedra in Figure S24A (blue peak) react to quickly form bromide-enriched $[PbX_6]^{4-}$ oc-

tahedra following the process in Figure 4D and more strongly coordinated Pb (or Pb_{SC}) atoms in Figure S24C-D (green peak) even when the O_2 gas is still present. As a result, the iodide deficiency in these PbI_2-rich perovskite films slows down I_3^- generation that is now barely visible in the deconvoluted high resolution I 3d core level spectra as demonstrated in Figure S25 after gas exposure. This means the rate of oxidation is decreased, which indicates that the rates of the complete active corrosion process and thus, perovskite degradation, are significantly lowered. We propose that the constant illumination with X-rays under O_2 generates a halide migration pathway along the grain boundaries within the perovskite so that the surrounding perovskite material acts as a feed to restore the bromide-enriched $[PbX_6]^{4-}$ octahedra as demonstrated in Figure 4E. Pb-I coordination bonds are more easily broken compared to Pb-Br coordination bonds, which means the non-illuminated perovskite material is a reservoir of mainly iodide anions. The fact that the anodic corrosion process is considerably slowed down and more stable Pb_{SC} atoms are directly formed.

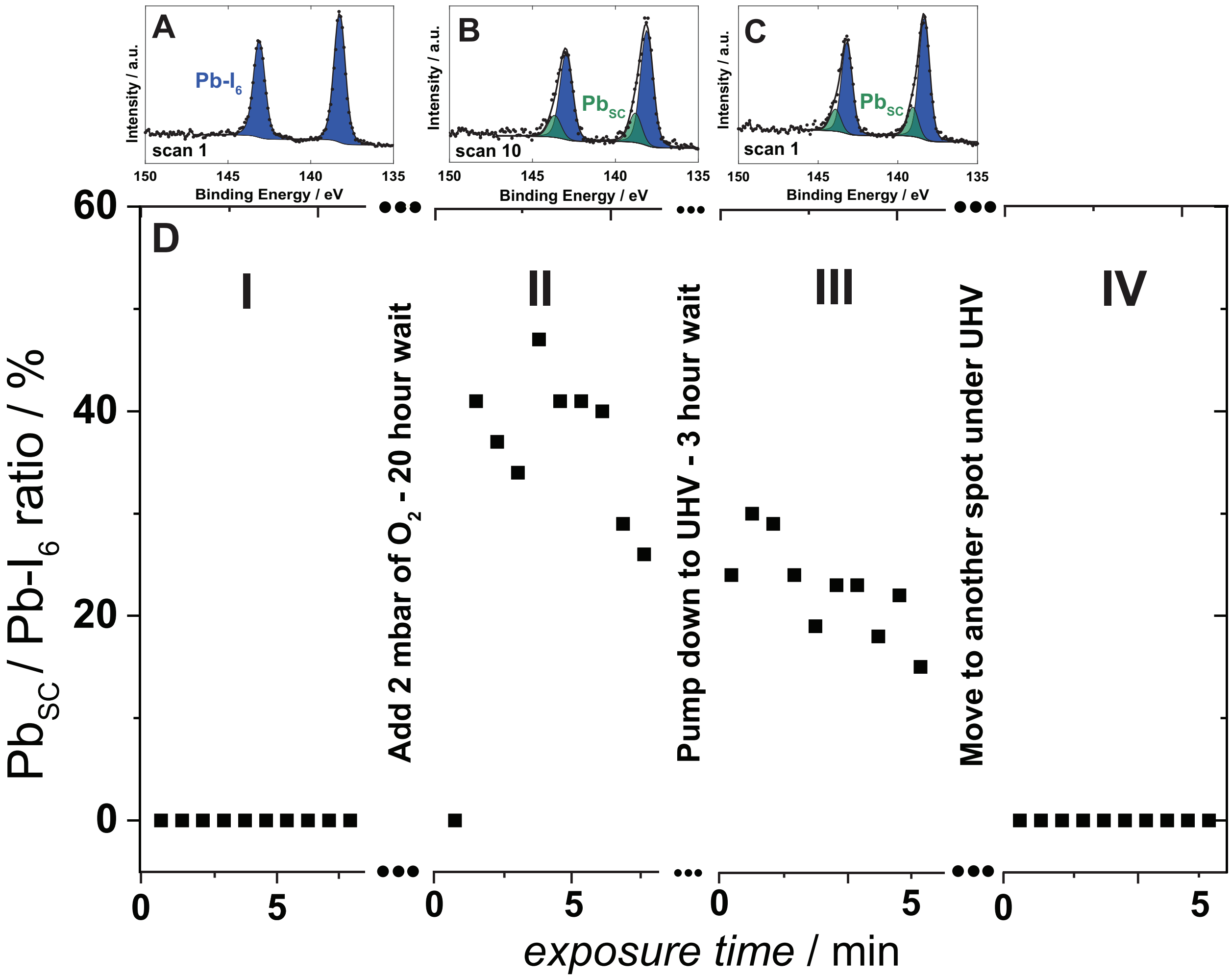

Figure S24 (A-C) High-resolution Pb 4f XPS data, and time dependencies for formation of Pb_{SC} for the PbI_2-rich/FAI-deficient perovskite $Cs_{.05}FA_{.79}MA_{.16}$ (A) under vacuum (only the $Pb\text{-}I_6$-like form observed); (B) same analysis spot during exposure to O_2/X-rays/white light Pb_{SC}/Pb_{total} decreases, (C) same analysis spot after return to UHV conditions continuous decrease; (D) Time dependencies of Pb_{SC}/Pb_{total} for conditions in (A-C). The last panel shows Pb_{SC}/Pb_{total} for a second analysis spot (under UHV conditions) that had been exposed to dry O_2 gas during (A-C), but not under X-ray illumination Pb 4f line shapes were unchanged from the as-deposited perovskite, with only one Pb form observed.

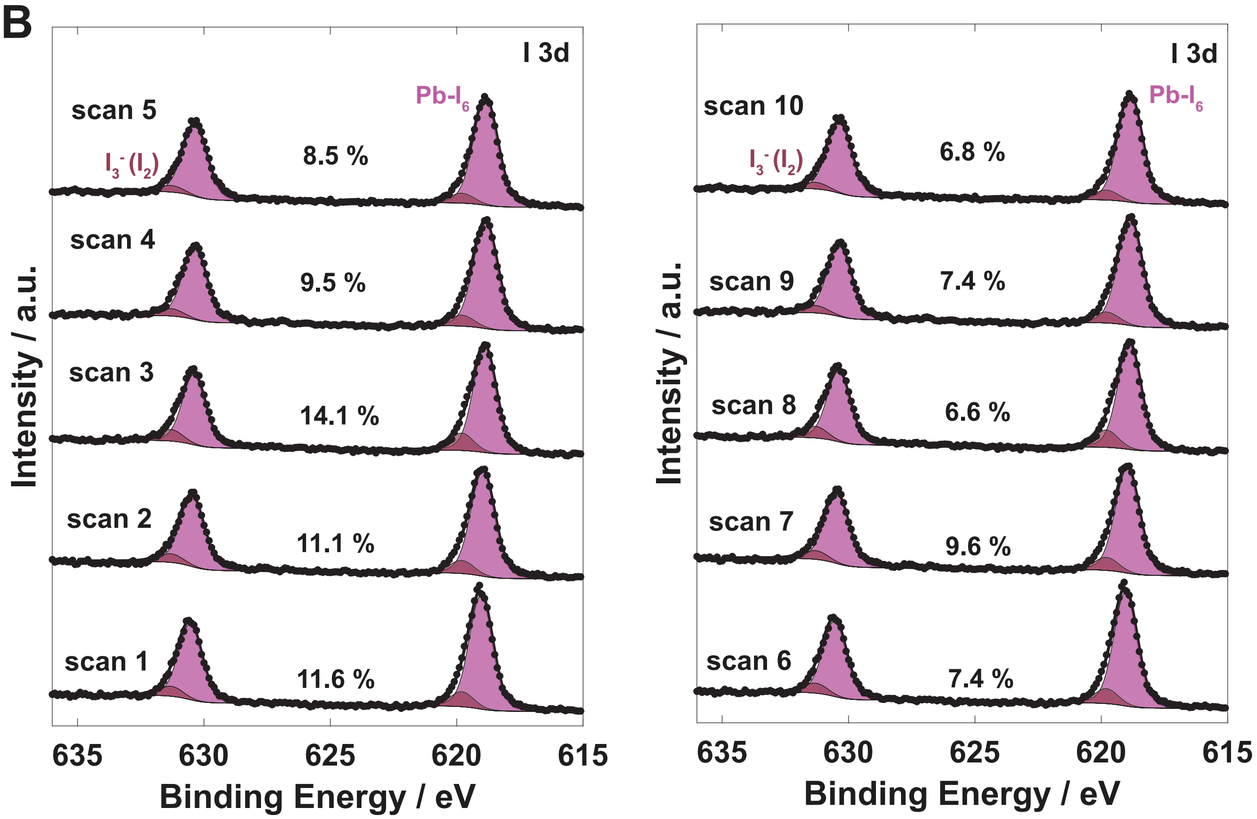

Figure S25 Individual (scan 1 to 10) I 3d high-resolution XPS core level spectra of a PbI_2-enriched triple cation perovskite film under UHV and white light after dry O_2 gas exposure. The percentages indicate the relative amount of oxidized iodide in the form of I_3^- (or chemisorbed I_2) compared to the total amount of iodide.

Notes and references

[1] Bahri, M.; Schnaider Tontini, F.; De Keersmaecker, M. L.; Ratcliff, E. L.; Armstrong, N. R.; Browning, N. D. FIB Sample Preparation and Low Dose STEM Characterisation Challenges of Hybrid Organic-inorganic Perovskite (HOIP) Solar Cells. *Microscopy Microanalysis* **2023**, *29*, 115–116.

[2] Philippe, B.; Park, B.-W.; Lindblad, R.; Oscarsson, J.; Ahmadi, S.; Johansson, E. M. J.; Rensmo, H. Chemical and Electronic Structure Characterization of Lead Halide Perovskites and Stability Behavior under Different Exposures A Photoelectron Spectroscopy Investigation. *Chemistry of Materials* **2015**, *27*, 1720–1731.

[3] Ning, S.; Zhang, S.; Sun, J.; Li, C.; Zheng, J.; Khalifa, Y. M.; Zhou, S.; Cao, J.; Wu, Y. Ambient Pressure X-ray Photoelectron Spectroscopy Investigation of Thermally Stable Halide Perovskite Solar Cells via Post-Treatment. *ACS Applied Materials and Interfaces* **2020**, *12*, 43705–43713.

[4] Kot, M.; Kegelmann, L.; Kobler, H.; Vorokhta, M.; Escudero, C.; Kus, P.; Smid, B.; Tallarida, M.; Albrecht, S.; Abate, A.; Matolinova, I.; Schmeisser, D.; Flege, J. I. In situ Near-Ambient Pressure X-ray Photoelectron Spectroscopy Reveals the Influence of Photon Flux and Water on the Stability of Halide Perovskite. *ChemSusChem* **2020**, *13*, 5722–5730.

[5] Il Jake Choi, J.; Ono, L. K.; Cho, H.; Kim, K. J.; Kang, H. B.; Qi, Y.; Park, J. Y. Pathways of Water-Induced Lead-Halide Perovskite Surface Degradation: Insights from In Situ Atomic-Scale Analysis. *ACS Nano* **2023**, *17*, 25679–25688.

[6] Steirer, K. X.; Schulz, P.; Teeter, G.; Stevanovic, V.; Yang, M.; Zhu, K.; Berry, J. J. Defect Tolerance in Methylammonium Lead Triiodide Perovskite. *ACS Energy Letters* **2016**, *1*, 360–366.

[7] Aristidou, N.; Eames, C.; Sanchez-Molina, I.; Bu, X.; Kosco, J.; Islam, M. S.; Haque, S. A. Fast Oxygen Diffusion and Iodide Defects Mediate Oxygen-Induced Degradation of Perovskite Solar Cells. *Nature Communications* **2017**, *8*, 15218.

[8] Ralaiarisoa, M. et al. Influence of X-Ray Irradiation During Photoemission Studies on Halide Perovskite-Based Devices. *Small Methods* **2023**, e2300458.

[9] Huang, W.; Sadhu, S.; Ptasinska, S. Heat- and Gas-Induced Transformation in $CH_3NH_3PbI_3$ Perovskites and Its Effect on the Efficiency of Solar Cells. *Chemistry of Materials* **2017**, *29*, 8478–8485.

[10] Tong, C.-J.; Li, L.; Liu, L.-M.; Prezhdo, O. V. Long Carrier Lifetimes in PbI2-Rich Perovskites Rationalized by Ab Initio Nonadiabatic Molecular Dynamics. *ACS Energy Letters* **2018**, *3*, 1868–1874.

[11] Jacobsson, T. J.; Correa-Baena, J. P.; Halvani Anaraki, E.; Philippe, B.; Stranks, S. D.; Bouduban, M. E.; Tress, W.; Schenk, K.; Teuscher, J.; Moser, J. E.; Rensmo, H.; Hagfeldt, A. Unreacted PbI2 as a Double-Edged Sword for Enhancing the Performance of Perovskite Solar Cells. *Journal of the American Chemical Society* **2016**, *138*, 10331–10343.

[12] De Keersmaecker, M.; Tirado, J.; Armstrong, N. R.; Ratcliff, E. L. Defect Quantification in Metal Halide Perovskites Anticipates Photoluminescence and Photovoltaic Performance. *ACS Energy Letters* **2024**, *9*, 243–252.

[13] Scofield, J. Hartree-Slater Subshell Photoionization Cross-Sections at 1254 and 1487 eV. *Journal of Electron Spectroscopy and Related Phenomena* **1976**, *8*, 129–137.

[14] Jablonski, A. Universal Energy Dependence of the Inelastic Mean Free Path. *Surface and Interface Analysis* **2004**, *20*, 317–321.

[15] Trotochaud, L.; Head, A. R.; Karslioglu, O.; Kyhl, L.; Bluhm, H. Ambient Pressure Photoelectron Spectroscopy: Practical Considerations and Experimental Frontiers. *Journal of Physics: Condensed Matter* **2017**, *29*, 053002.

[16] Bluhm, H. Photoelectron Spectroscopy of Surfaces Under Humid Conditions. *Journal of Electron Spectroscopy and Related Phenomena* **2010**, *177*, 71–84.

[17] Tao, F.; Nguyen, L. Interactions of Gaseous Molecules with X-ray Photons and Photoelectrons in AP-XPS Study of Solid Surface in Gas Phase. *Physical Chemistry Chemical Physics* **2018**, *20*, 9812–9823, (Feng).

[18] Kjærvik, M. Applications of Near-Ambient Pressure X-ray Photoelectron Spectroscopy in Life Science and Materials Research. Thesis, 2021.

[19] Pederson, L. Two-Dimensional Chemical-State Plot For Lead Using XPS. *Journal of Electron Spectroscopy and Related Phenomena* **1982**, *28*, 203–209.

[20] Scheidt, R. A.; Kamat, P. V. Temperature-Driven Anion Migration in Gradient Halide Perovskites. *Journal of Chemical Physics* **2019**, *151*, 134703.

[21] Kerner, R. A.; Xu, Z.; Larson, B. W.; Rand, B. P. The Role of Halide Oxidation in Perovskite Halide Phase Separation. *Joule* **2021**, *5*, 2273–2295.

[22] Dillard, J. G.; Moers, H.; Klewe-Nebenius, H.; Kirch, G.; Pfennig, G.; Ache, H. J. An X-ray Photoelectron and Auger Electron Spectroscopic Study of the Adsorption of Molecular Iodine on Uranium Metal and Uranium Dioxide. *Journal of Physical Chemistry* **1984**, *88*, 4104–4111.

[23] Mundt, L. E.; Tong, J.; Palmstrom, A. F.; Dunfield, S. P.; Zhu, K.; Berry, J. J.; Schelhas, L. T.; Ratcliff, E. L. Surface-Activated Corrosion in TinLead Halide Perovskite Solar Cells. *ACS Energy Letters* **2020**, *5*, 3344–3351.

[24] Xu, Z.; Kerner, R. A.; Harvey, S. P.; Zhu, K.; Berry, J. J.; Rand, B. P. Halogen Redox Shuttle Explains Voltage-Induced Halide Redistribution in Mixed-Halide Perovskite Devices. *ACS Energy Letters* **2022**, *8*, 513–520.

[25] Dupin, J.-C.; Gonbeau, D.; Vinatier, P.; Levasseur, A. Systematic XPS Studies of Metal Oxides, Hydroxides and Peroxides. *Physical Chemistry Chemical Physics* **2000**, *2*, 1319–1324.

[26] Bruijnaers, B. J.; Schiepers, E.; Weijtens, C. H. L.; Meskers, S. C. J.; Wienk, M. M.; Janssen, R. A. J. The Effect of Oxygen on the Efficiency of Planar PIN Metal Halide Perovskite Solar Cells with a PEDOT:PSS Hole Transport Layer. *Journal of Materials Chemistry A* **2018**, *6*, 6882–6890.

[27] Jung, H. J.; Kim, D.; Kim, S.; Park, J.; Dravid, V. P.; Shin, B. Stability of Halide Perovskite Solar Cell Devices: In Situ Observation of Oxygen Diffusion under Biasing. *Advanced Materials* **2018**, *30*, e1802769.

[28] Senocrate, A.; Acartürk, T.; Kim, G. Y.; Merkle, R.; Starke, U.; Grätzel, M.; Maier, J. Interaction of Oxygen with Halide Perovskites. *Journal of Materials Chemistry A* **2018**, *6*, 10847–10855.

[29] Li, B.; Dai, Q.; Yun, S.; Tian, J. Insights Into Iodoplumbate Complex Evolution of Precursor Solutions for Perovskite Solar Cells: From Aging to Degradation. *Journal of Materials Chemistry A* **2021**, *9*, 6732–6748.

[30] Sharenko, A.; Mackeen, C.; Jewell, L.; Bridges, F.; Toney, M. F. Evolution of Iodoplumbate Complexes in Methylammonium Lead Iodide Perovskite Precursor Solutions. *Chemistry of Materials* **2017**, *29*, 1315–1320.

[31] Wakamiya, A.; Endo, M.; Sasamori, T.; Tokitoh, N.; Ogomi, Y.; Hayase, S.; Murata, Y. Reproducible Fabrication of Efficient Perovskite-based Solar Cells: X-ray Crystallographic Studies on the Formation of CH3NH3PbI3 Layers. *Chemistry Letters* **2014**, *43*, 711–713.

[32] Avval, T. G.; Chatterjee, S.; Hodges, G. T.; Bahr, S.; Dietrich, P.; Meyer, M.; ThiSSen, A.; Linford, M. R. Oxygen Gas, O2(g), by Near Ambient Pressure XPS. *Surface Science Spectra* **2019**, *26*, 014021.

[33] He, J.; Fang, W. H.; Long, R.; Prezhdo, O. V. Why Oxygen Increases Carrier Lifetimes but Accelerates Degradation of CH3NH3PbI3 under Light Irradiation: Time-Domain Ab Initio Analysis. *Journal of the American Chemical Society* **2020**, *142*, 14664–14673.

[34] He, J.; Fang, W. H.; Long, R.; Prezhdo, O. V. Superoxide/Peroxide Chemistry Extends Charge Carriers' Lifetime but Undermines Chemical Stability of CH3NH3PbI3 Exposed to Oxygen: Time-Domain ab Initio Analysis. *Journal of the American Chemical Society* **2019**, *141*, 5798–5807.

[35] Hippler, M. Photochemical Kinetics: Reaction Orders and Analogies with Molecular Beam Scattering and Cavity Ring-Down Experiments. *Journal of Chemical Education* **2003**, *80*, 1074.

[36] Aristidou, N.; Sanchez-Molina, I.; Chotchuangchutchaval, T.; Brown, M.; Martinez, L.; Rath, T.; Haque, S. A. The Role of Oxygen in the Degradation of Methylammonium Lead Trihalide Perovskite Photoactive Layers. *Angewandte Chemie International Edition* **2015**, *54*, 8208–8212.

[37] Mundt, L. E.; Schelhas, L. T. Structural Evolution During Perovskite Crystal Formation and Degradation: In Situ and Operando XRay Diffraction Studies. *Advanced Energy Materials* **2019**, *10*, 1903074.

[38] Boyd, C. C. et al. Overcoming Redox Reactions at Perovskite-Nickel Oxide Interfaces to Boost Voltages in Perovskite Solar Cells. *Joule* **2020**, *4*, 1759–1775.

[39] Hoke, E. T.; Slotcavage, D. J.; Dohner, E. R.; Bowring, A. R.; Karunadasa, H. I.; McGehee, M. D. Reversible Photo-induced Trap Formation in Mixed-Halide Hybrid Perovskites for Photovoltaics. *Chemical Science* **2015**, *6*, 613–617.

[40] Barker, A. J.; Sadhanala, A.; Deschler, F.; Gandini, M.; Senanayak, S. P.; Pearce, P. M.; Mosconi, E.; Pearson, A. J.; Wu, Y.; Srimath Kandada, A. R.; Leijtens, T.; De Angelis, F.; Dutton, S. E.; Petrozza, A.; Friend, R. H. Defect-Assisted Photoinduced Halide Segregation in Mixed-Halide Perovskite Thin Films. *ACS Energy Letters* **2017**, *2*, 1416–1424.

[41] BenavidesGarcia, M.; Balasubramanian, K. Bond Energies, Ionization Potentials, and the SingletTriplet Energy Separations of SnCl2, SnBr2, SnI2, PbCl2, PbBr2, PbI2, and Their Positive Ions. *The Journal of Chemical Physics* **1994**, *100*, 2821–2830.

[42] Armstrong, D. A.; Huie, R. E.; Koppenol, W. H.; Lymar, S. V.; Merényi, G.; Neta, P.; Ruscic, B.; Stanbury, D. M.; Steenken, S.; Wardman, P. Standard Electrode Potentials Involving Radicals in Aqueous Solution: Inorganic Radicals (IUPAC Technical Report). *Pure and Applied Chemistry* **2015**, *87*, 1139–1150.

[43] Morgan, W. E.; Van Wazer, J. R.; Stec, W. J. Inner-Orbital Photoelectron Spectroscopy of the Alkali Metal Halides, Perchlorates, Phosphates, and Pyrophosphates. *Journal of the American Chemical Society* **1973**, *95*, 751–755.
